# Liver Transcriptome Dynamics During Hibernation Are Shaped by a Shifting Balance Between Transcription and RNA Stability

**DOI:** 10.3389/fphys.2021.662132

**Published:** 2021-05-21

**Authors:** Austin E. Gillen, Rui Fu, Kent A. Riemondy, Jennifer Jager, Bin Fang, Mitchell A. Lazar, Sandra L. Martin

**Affiliations:** ^1^RNA Bioscience Initiative, University of Colorado School of Medicine, Aurora, CO, United States; ^2^Division of Endocrinology, Diabetes, and Metabolism, Department of Medicine, and The Institute for Diabetes, Obesity and Metabolism, Perelman School of Medicine, University of Pennsylvania, Philadelphia, PA, United States; ^3^Division of Endocrinology, Diabetes, and Metabolism, Department of Genetics, and The Institute for Diabetes, Obesity and Metabolism, Perelman School of Medicine, University of Pennsylvania, Philadelphia, PA, United States; ^4^Department of Cell and Developmental Biology, University of Colorado School of Medicine, Aurora, CO, United States

**Keywords:** A-to-I RNA editing, ARE-binding proteins, alternative splicing, hepatocyte, Ictidomys tridecemlineatus

## Abstract

Hibernators dramatically lower metabolism to save energy while fasting for months. Prolonged fasting challenges metabolic homeostasis, yet small-bodied hibernators emerge each spring ready to resume all aspects of active life, including immediate reproduction. The liver is the body’s metabolic hub, processing and detoxifying macromolecules to provide essential fuels to brain, muscle and other organs throughout the body. Here we quantify changes in liver gene expression across several distinct physiological states of hibernation in 13-lined ground squirrels, using RNA-seq to measure the steady-state transcriptome and GRO-seq to measure transcription for the first time in a hibernator. Our data capture key timepoints in both the seasonal and torpor-arousal cycles of hibernation. Strong positive correlation between transcription and the transcriptome indicates that transcriptional control dominates the known seasonal reprogramming of metabolic gene expression in liver for hibernation. During the torpor-arousal cycle, however, discordance develops between transcription and the steady-state transcriptome by at least two mechanisms: 1) although not transcribed during torpor, some transcripts are unusually stable across the torpor bout; and 2) unexpectedly, on some genes, our data suggest continuing, slow elongation with a failure to terminate transcription across the torpor bout. While the steady-state RNAs corresponding to these read through transcripts did not increase during torpor, they did increase shortly after rewarming despite their simultaneously low transcription. Both of these mechanisms would assure the immediate availability of functional transcripts upon rewarming. Integration of transcriptional, post-transcriptional and RNA stability control mechanisms, all demonstrated in these data, likely initiate a serial gene expression program across the short euthermic period that restores the tissue and prepares the animal for the next bout of torpor.

## Introduction

**FIGURE 1:**
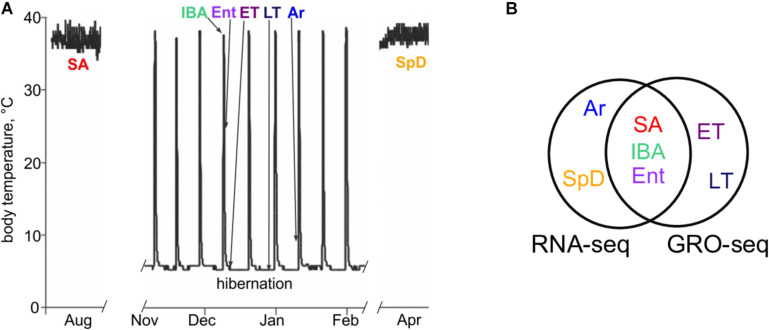
Physiological groups analyzed for differential gene expression in liver using RNA-seq and GRO-seq. **(A)** Schematic shows Tb characteristics of animals in active (SA and SpD) vs. hibernation seasons (IBA, Ent, ET, LT, Ar). RNA-seq data were collected from *n* = 5 individuals in SA, IBA, Ent, Ar and SpD. Groseq data were collected for *n* = 3 individuals in SA, IBA, Ent, ET and LT (see Supplementary Table 1 for metadata). **(B)** Venn diagram shows common and unique sample groups studied by RNA-seq and GRO-seq.

Hibernation is an adaptive strategy that enables survival of prolonged food deprivation. Each fall, 13-lined ground squirrels cease activity and disappear into their hibernacula. The animals do not eat or drink for several months, instead fueling their metabolism with endogenous stores, largely fat, that were acquired during the previous summer. Metabolic and heart rates are profoundly reduced, allowing body temperature (Tb) to fall to just above freezing where it remains for many days. By spending most of the fall and winter in this depressed metabolic state known as torpor, the animals save up to 90% of the energy they would have needed to remain active throughout their half year of hibernation. Paradoxically, however, the majority of the energy budget for the season of hibernation is consumed when torpor is periodically interrupted for an interbout arousal (IBA); after several days to even weeks in torpor, hibernating ground squirrels spontaneously reactivate metabolism to restore euthermic Tb for approximately 12 h. Thus, hibernation is not a static quiescent state, but rather a dynamic pattern of seasonal heterothermy accompanied by prolonged fasting (Figure 1, see also Carey et al., 2003; van Breukelen and Martin, 2015, for review). These rapid and dramatic shifts in temperature as well as oxygen delivery and utilization – and thus metabolism – present multiple challenges to the maintenance of homeostasis at every level of the hibernator’s biological organization.

The purpose of the energetically expensive arousals from torpor is not known, although, at least in lemurs, they appear to be compelled only in animals exhibiting torpid Tb < 30°C (Dausmann et al., 2004). Clearly rates of biochemical reactions slow as Tb decreases during torpor, and differences in the thermal sensitivity of various reactions throughout the body leads to widespread changes, both increases and decreases, in the relative abundances of metabolites, transcripts and proteins across the torpor bout; the short, warm arousal periods are also highly dynamic, with changes between initial rewarming through the beginning of cooling when the next bout of torpor begins (Nelson et al., 2009; Epperson et al., 2011; Hindle et al., 2011, 2014; Jani et al., 2012; Grabek et al., 2015a; Bogren et al., 2017; D’Alessandro et al., 2017; Regan et al., 2019; Rice et al., 2020). While rewarming restores the slowed cellular processes and biochemical reaction rates of torpor in every system in the body, the detailed molecular mechanisms orchestrating these dynamics remain to be fully elucidated (see van Breukelen and Martin, 2015, and references therein).

Liver is a key organ for maintaining metabolic homeostasis throughout the body. It is especially important during feeding and fasting, because hepatocytes, the main cell type of the liver, process, metabolize and repackage macronutrients from food and detoxify xenobiotics. Although no comparison to the half-year long fast of hibernating 13-lined ground squirrels, mice fasted for 21 h have a robust transcriptional response to fasting and refeeding that involves approximately 30% of the genes expressed in liver (Chi et al., 2020). But hepatocytes also produce the bulk of plasma proteins and play significant supporting roles in other crucial physiological processes including blood volume control, immune system function, and endocrine growth factor signaling (Trefts et al., 2017). Thus, metabolic depression and low Tb during torpor present additional challenges to liver and whole animal homeostasis during hibernation. In addition to the expected effects of the prolonged seasonal fast on liver gene expression, the enhanced resistance to ischemia-reperfusion injury during hibernation (Lindell et al., 2005) suggests a role for dynamically regulated gene expression in orchestrating a protected phenotype.

Despite the liver’s central role in metabolism, it being the source of the first differentially expressed gene in a hibernator (Srere et al., 1992), and the discovery that transcription all but ceases at the low Tb of torpor but resumes during each rewarming (van Breukelen and Martin, 2002), we still have incomplete knowledge of differential gene regulation in the liver during the seasonal and torpor-arousal cycles of hibernation. While many studies have considered gene expression changes in a small number of specific genes, broad screens with the potential to discover novel or unexpected hibernation genes are lacking. Previous efforts used custom microarrays (Williams et al., 2005) or bead arrays (Yan et al., 2008), as well as early high-throughput sequencing approaches for species lacking robust genomes (Bogren et al., 2017; Nespolo et al., 2018), all of which limited DE analyses to a set of almost exclusively protein coding genes previously described in non-hibernators.

To begin to overcome these limitations and gain further insight into liver gene expression dynamics in hibernation, we collected high-quality, paired-end, strand-specific RNA-seq data from five individuals in each of five physiological states across the hibernator’s year. These samples distinguish seasonal and torpor-arousal cycle variation. In addition, reads were mapped to a greatly enhanced ground squirrel genome and annotation for quantification. The genome approaches chromosome-level integrity and includes 42,881 previously identified, newly annotated and novel genes (Fu et al., 2021). We further assess the role of transcriptional control in determining the observed differential gene expression using GRO-seq data (Core et al., 2008), collected for the first time from a hibernator. Taken together these two datasets demonstrate that transcriptional control dominates the seasonal gene expression changes, while both transcriptional and post-transcriptional mechanisms are clearly involved in gene expression dynamics across the torpor-arousal cycle. The RNA-seq data were further analyzed for A-to-I RNA editing and differential splicing, both previously shown to occur in brain (Riemondy et al., 2018; Fu et al., 2021), demonstrating that both cold-associated ADAR-mediated RNA editing and differential splicing also occur in liver. As in brain (Fu et al., 2021), these liver data demonstrate the necessity and value of carefully timed samples for use in this type of hibernation study and provide both a resource and a roadmap to guide future work.

## Results

Liver tissue was collected from 38 individuals representing seven physiological states across the hibernator’s year (Figure 1 and Supplementary Table 1). RNA was isolated from 25 of these livers and converted to strand-specific RNA-seq libraries to quantify the steady-state transcriptome across five physiological states: two that bracketed the hibernation season (SA and SpD) and three that captured the torpor-arousal cycles of hibernation (IBA, Ent and Ar). Nuclei isolated from 15 livers in five states were chosen to directly examine transcription across the torpor-arousal cycle (IBA, Ent, ET and LT, compared to the non-hibernating SA) using GRO-seq (Core et al., 2008). Two of the livers were used to collect both types of data, SA30 and Ent64.

### RNA-Seq Data Analysis

The RNA-seq data set comprised 33.2 ± 3.3 million high-quality, strand-specific readpairs (Supplementary Table 1) from each of five biological replicates from each state as depicted in Figure 1. The sequences were first aligned to mtDNA, which were elevated in SA (Supplementary Table 1). This finding likely reflects the critical role of liver mitochondria in converting dietary nutrients into fatty acids as the animals fatten in preparation for winter hibernation (Lanaspa et al., 2015). The remaining, non-mitochondrial reads were processed for gene-based analyses of differential expression (see section “Materials and Methods”).

**FIGURE 2:**
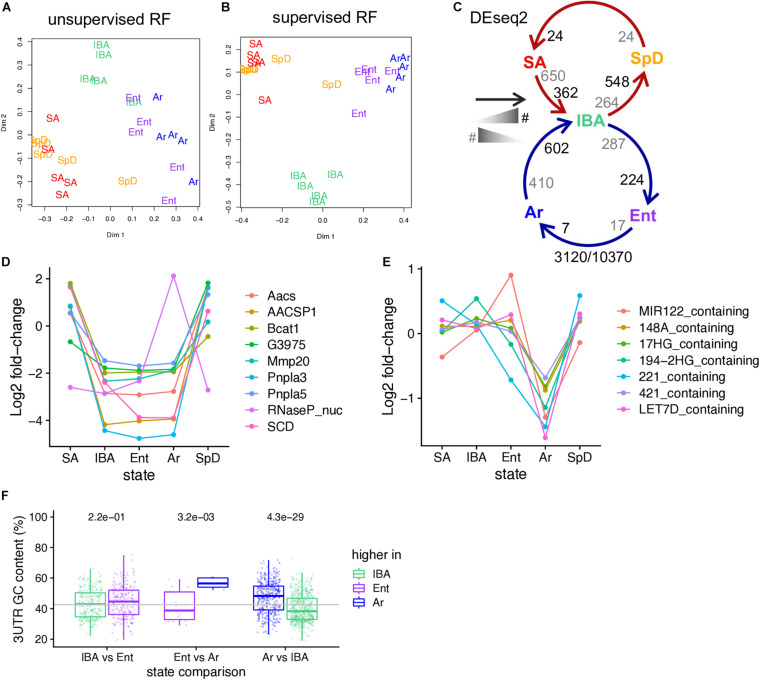
RNA-seq reveals changes in the relative abundance of liver steady-state RNAs as a function of hibernation physiology. Two-dimensional scaling plots obtained by unsupervised **(A)** or supervised **(B)** clustering of RNA-seq pass-filter reads by random forest; each label represents one individual (groups are as defined in Figure 1). **(C)** Schematic of seasonal (SpD - > SA - > IBA) and hibernation (IBA - > Ent - > Ar) cycles showing the number of DE genes across each pairwise transition. As indicated in the schematic legend (left), the number of DE genes increased in the state closest to the head or the tail of the arrow are enumerated in black and gray, respectively. Numbers below report the number of DE genes/the number of pass-filter liver genes evaluated. Line plots of **(D)** 9 liver DE genes exhibiting log2 fold change > 5; **(E)** Differentially expressed microRNA precursor genes. **(F)** Boxplot showing the GC-content of DE genes (dots) across the torpor-arousal cycle. Each of the three possible pairwise comparisons comprising the torpor-arousal cycle depicted in panel C are indicated below, genes elevated in each state of the pair are plotted by color (legend). See also Supplementary Data Sheet 3, Supplementary Table 2.

Unsupervised random forest (RF) clustering of the samples using the log transformed pseudocounts from all pass-filter nuclear genes reveals clear separation between the homeothermic active animals (SpD and SA) and the heterothermic hibernators (IBA, Ent and Ar), demonstrating a prominent effect of hibernation physiology on the liver transcriptome (Figure 2A). Among the winter hibernation groups, IBA was the most distinct, although all 5 groups were cleanly separated when variable selection was used to optimize group separation (Figure 2B). Consistent with the RF results, DESeq2 analysis revealed that 3,120 of the 10,370 pass-filter genes evaluated (∼30%), were differentially expressed (i.e., *q* < 0.001, DE, Figure 2C) among the five states. Hundreds of DE genes distinguished the homeotherms from the heterotherms (compare SA or SpD to IBA, Figure 2C), and the IBA hibernators from both the Ent and Ar hibernators (Figure 2C). It is noteworthy that the majority of DE genes (approximately two-thirds) that distinguished the three hibernating states from non-hibernating euthermic states were decreased in the hibernators. This finding suggests a large seasonal shift with a general down-regulation of the genes comprising the liver transcriptome in hibernation compared to active, feeding animals. Far fewer liver genes were DE between animals that had recently emerged (SpD) from hibernation and those preparing to immerge (SA) into hibernation. The smallest number of DE genes differentiated Ent from Ar hibernators (Figure 2C), despite this being the longest time segment in the torpor-arousal cycle (Figure 1A). The largest pairwise fold-changes, on the order of 200-fold (| log2FC| > 7.79) were found among genes increased in both homeotherms compared to IBA and increased during Ar compared to IBA (Supplementary Table 2).

Exploring the genes with the highest fold changes reveals that nine transcripts with log2 fold-changes greater than five are found in the data. Most of these are protein-coding genes which were seasonally decreased throughout hibernation. Just the RNaseP_RNA had a different pattern, which was a dramatic increase across the torpor bout (Figure 2D). Many of the higher fold changes across the pairwise transitions are also MIR_containing, i.e., the longer primary RNA precursors of functional miRNAs: all of these decrease across the torpor bout (Figure 2E). Similar to brain (Fu et al., 2021), transcripts increased in the cold (i.e., higher in Ar than either Ent or IBA) were also biased for higher GC content, as were transcripts elevated in IBA compared to SpD (Figure 2F).

**FIGURE 3:**
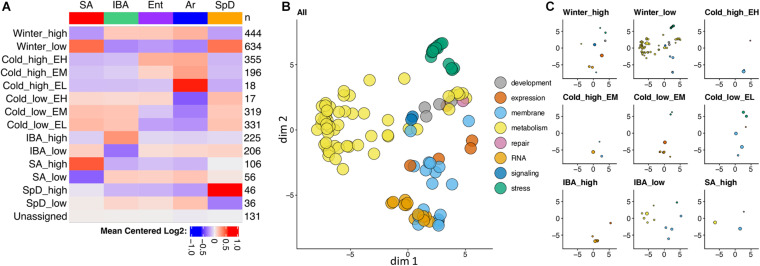
Seasonally decreased metabolic genes dominate differential gene expression in hibernator liver. **(A)** Heatmap summary of RNA-seq co-expression data for 3,120 DE genes in liver. Colors indicate the relative abundance of steady-state RNA for each physiological state. Pattern names (left) and the number of genes (right) in each co-expression cluster are indicated. **(B,C)** Gene enrichment categories in the DE genes. Two-dimensional scaling plots for gene enrichments based on similarity of terms, using REVIGO **(B)** and then replotted in panel **(C)** after segregating by co-expression pattern and adjusting so that circle diameters are proportional to the number of DE genes in each enrichment category. Colors group enrichments to the indicated broad categories: expression = regulation of gene expression, repair = DNA repair; RNA = RNA splicing and processing, and stress = stress response and signaling. In panel **(C)**, co-expression clusters with < 2 significant enrichments are excluded. See also Supplementary Figure 2 and Supplementary Table 3.

To better understand liver gene expression dynamics during hibernation, we used WGCNA (Langfelder and Horvath, 2008) to first cluster the DE genes by their relative transcript abundance pattern across the five physiological groups and then correlate those co-expression patterns with phenotypic characteristics (Supplementary Figure 1). Consistent with the findings from RF and pairwise analyses, we observed strong correlations (>0.8) of cluster patterns to heterothermy, Tb and IBA. Because closer inspection of individual genes revealed many exhibited gene expression dynamics inconsistent with their WGCNA assignments (see also, Botía et al., 2017; Fu et al., 2021), we defined 14 most-common DE patterns in the data and re-assigned the 3,120 DE genes to their best-fit cluster. This process placed 95.8% of the DE genes into one of 14 dynamic patterns (Figure 3A and Supplementary Figure 2).

Strong effects of hibernation season and Tb on liver transcriptome dynamics dominated the liver co-expression patterns. More than one third of the DE genes were increased (Winter_high) or decreased (Winter_low) throughout the several months of hibernation (IBA, Ent, Ar) compared to active animals (SA, SpD). And, nearly 40% were altered by temperature, either increasing (Cold_high) or decreasing (Cold_low) by the end of the torpor bout (Ar) compared to 2–3 h after rewarming (IBA), with different relative abundances at the end of the euthermic period as the animals reentered torpor (EH, EM, EL, for Ent high, medium or low, respectively). There were relatively few genes in the two clusters with maximal change across the torpor bout, i.e., Cold_low_EH or Cold_high_EL, consistent with the RF clustering in Figure 2A and the pairwise analysis in Figure 2C. Another 14% of DE genes were increased or decreased exclusively early in the euthermic period between torpor bouts (IBA_high, IBA_low). Finally, small sets of DE genes altered uniquely in one of the homeothermic periods, SA or SpD, were also apparent in the data. Further insight comes from analysis of enriched gene ontology terms in these clusters. Not unexpectedly for liver, gene enrichments exhibited an overarching dominance of terms related to metabolism (yellow circles in Figure 3B), with the vast majority of these segregating to the Winter_low co-expression group (Figure 3C, Supplementary Figure 2, and Supplementary Table 3), consistent with the reduced need to process nutrient influx during the long winter fast.

The 214 genes that increased in the cold (Cold_high_EM + Cold_high_EL) in these RNA-seq data were particularly intriguing given that transcription in ground squirrel liver was shown previously to all but cease (van Breukelen and Martin, 2002) at the low Tb of torpor. Because RNA-seq measures the relative abundance of transcripts at steady-state, differences in transcription, stability or even polyadenylation (Grabek et al., 2015a) could account for the observed transcriptome differences between animals just beginning (Ent) vs. those just ending (Ar) a torpor bout. To directly assess the role of transcription in the observed transcriptome dynamics across the torpor-arousal cycle, we prepared GRO-seq libraries from liver in four stages of hibernation: IBA, Ent, and early and late torpor (ET and LT, respectively, Figure 1), and compared them to a non-hibernating SA group. GRO-seq is a high-throughput sequencing adaptation (Wissink et al., 2019) of the run-off assay used previously in hibernating ground squirrel liver (van Breukelen and Martin, 2002). Isolated nuclei were incubated to extend RNA polymerases that were pre-initiated and competent for elongation at the time of tissue collection. These newly transcribed RNAs were isolated, converted to sequencing libraries and sequenced such that only genes harboring active RNA polymerase across our various hibernation states were recovered in the GRO-seq libraries. The gene-based read counts from these libraries provide an accurate measure of relative transcription, in contrast to the multiple factors that govern the relative abundance of transcripts in steady-state RNA, i.e., as measured above by RNA-seq.

### GRO-Seq Data Analysis

**FIGURE 4:**
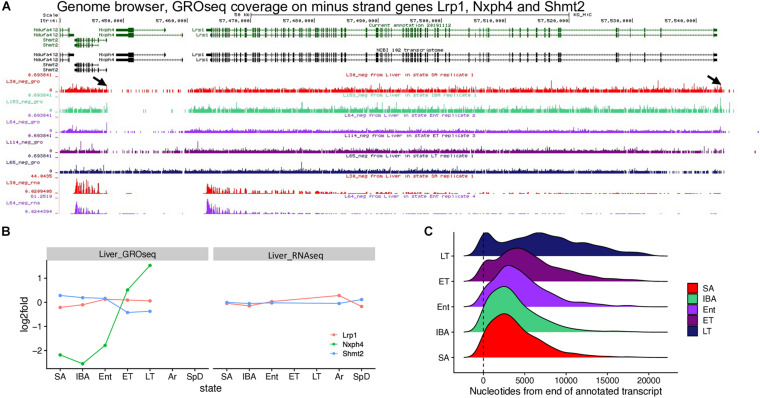
GRO-seq reveals transcriptional run-on and failure to terminate during torpor. **(A)** Browser view shows 13-lined ground squirrel (HiC_Itri_2) genomic region with Nxph4 downstream of Lrp1 and upstream of Shmt2, all transcribed from the minus strand, along with GRO-seq coverage from one representative sample from each state across this region and RNAseq from the common SA and Ent samples. Arrows mark paused polymerase near transcriptional start sites (promoter proximal pause) for Lrp1 and Shmt2. **(B)** Line graphs plot log2 fold change for these three genes in GRO-seq and RNA-seq data. **(C)** Distribution of fstitch segments called in GRO-seq data for each hibernation state, relative to the position of the 3′ end (at 0, ± 20kb) of annotated genes. The 3′ ends included in the analysis were required to have fstitch annotations called in all 5 states and these annotations were required to overlap the annotated 3′ end. RNA polymerase is known to accumulate at both the 5′ and 3′ ends of transcripts (Core et al., 2008; Glover-Cutter et al., 2008). See also Supplementary Figures 3, 6, and 13.

The GRO-seq dataset comprised 32.2 ± 3.7 million, strand-specific single-end reads from three biological replicates representing each of the five physiological states. After adapter removal, quality trimming and a length filter, 14.9 ± 3.4 million reads/sample remained; of these, 10.2 ± 2.2 million reads/sample aligned to the HiC genome (Supplementary Table 1). Initially, we quantified these reads using the same transcript annotation (Fu et al., 2021) as used for the liver RNA-seq analysis described above. Because previous studies of torpid hibernators had demonstrated depressed transcription at low Tb, we were astonished to find large numbers of DE genes that increased in ET compared to Ent, LT compared to ET, or LT compared to IBA (e.g., Nxph4 in Figures 4A,B; Supplementary Figure 3A). Inspection of the GRO-seq coverage on genes increased in LT compared to ET using the genome browser revealed they were found adjacent to an upstream, actively transcribed gene (Figures 4A,B; Supplementary Figures 3B–E), suggesting that elongation by RNA polymerase on liver genes can continue slowly throughout the torpor bout in concert with a failure to terminate transcription.

Metaplots comparing RNA-seq data with GRO-seq data by state lend further credence to the view that transcription fails to terminate during torpor. In contrast to the RNA-seq data, where read coverage near the 3′ end of transcripts abruptly decreased at the transcription termination site (TTS) independent of physiological status (Supplementary Figure 3F), elongating RNA polymerase captured by GRO-seq extended further and further 3′ of the TTS as animals began to cool during Ent and then progressed through the torpor bout (compare SA and IBA to Ent, ET and LT in Supplementary Figure 3G). Therefore, we used Fstitch (Azofeifa et al., 2017) on the GRO-seq data to reassess transcription units for 13-lined ground squirrel liver, analyzing each state separately. Comparison of the Fstitch transcribed segments among the five physiological states revealed a genomewide 3′ shift of elongating PolII on a subset of liver genes during torpor (Figure 4C), as seen by the second peak downstream of the TTS as animals begin to cool when re-entering torpor and its movement and broadening further to the right of the TTS as animals progressed from Ent -> ET -> LT in the torpor-arousal cycle of hibernation.

**FIGURE 5:**
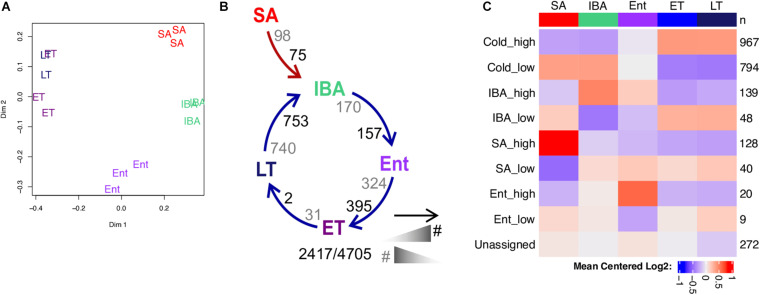
Differentially transcribed genes in liver during hibernation captured by GRO-seq. **(A)** Two-dimensional scaling plot shows unsupervised random forest clustering of individuals based on the full GRO-seq dataset. **(B)** Numbers of differentially transcribed genes between pairs of sequential stages in the torpor-arousal cycles of hibernation, compared to summer. As indicated in the legend below, the number of differentially transcribed genes increased in the state closest to the head or the tail of the arrow are enumerated in black and gray, respectively. Numbers below are the number DE/total fstitch gene-body transcription units. **(C)** Heatmap summarizes the GRO-seq coexpression clusters with the pattern indicated on the left and number of genes in each cluster indicated on the right. See also Supplementary Figure 5 and Supplementary Table 4.

Because of the features of transcription during torpor described above, to more accurately assess bona fide transcriptional changes associated with hibernation, we re-quantified the GRO-seq reads using a re-defined transcript annotation based on these Fstitch segments. We also removed the first 500nt downstream of the transcription start site (TSS, Vihervaara et al., 2017) and considered only the non-overlapping genes for this quantification. As with the steady-state transcriptome analyzed by RNA-seq, random forests analysis of the remaining 4,705 pass-filter GRO-seq genes revealed a strong effect of hibernation physiology on transcription. The SA, IBA and Ent groups were all well-separated from one another, and from the closely juxtaposed low Tb groups, ET and LT (Figure 5A). DESeq2 analysis of the GRO-seq data revealed sequential pairwise differences across the physiological states that were consistent with the random forest clustering. Strikingly, over half (2,417 or 56%) of the GRO-seq genes analyzed by DESeq2 were DE among the five physiological groups, with the largest number of pairwise differences distinguishing the torpid animals from the warmer stages of the torpor-arousal cycle (i.e., Ent vs. ET and LT vs. IBA, Figure 5B). Although the small sample size limits the utility of WGCNA, we see once again that the highest correlations (>0.8) between transcript co-expression patterns and phenotypic characteristics are to heterothermy, SA and IBA (Supplementary Figure 4). Re-clustering the GRO-seq genes by these traits captures 88% of the DE genes in just six patterns with the vast majority (73%) having the pattern Cold_high_EM or Cold_low_EM (Figure 5C and Supplementary Figure 5). Among these co-expression patterns in the GRO-seq data, only the Cold_high pattern was enriched for genes with extended 3′ ends based on the fstitch annotation, 62%, compared to 56% of the GRO-seq genes overall (Supplementary Table 4). Gene enrichment analysis revealed a dominance of mRNA splicing, processing and export among the genes with the Cold_high_EM pattern, whereas genes in the Cold_low_EM cluster were enriched for a metabolic activity, oxidation-reduction (Supplementary Figure 5 and Supplementary Table 3).

### Comparison of Genes DE in Both RNA-Seq and GRO-Seq Datasets

**FIGURE 6:**
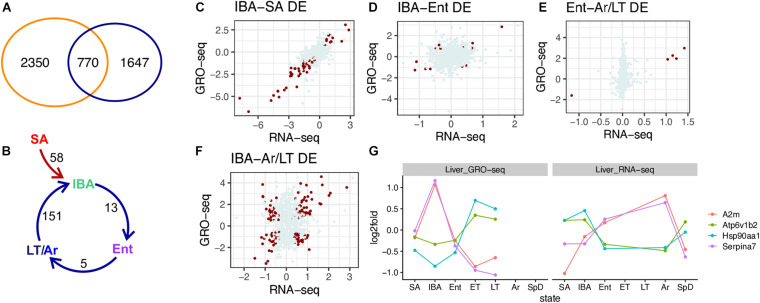
Comparison of DE genes identified by RNA-seq and GRO-seq. **(A)** Venn diagram indicates the number of DE genes in just RNA-seq (blue), just GRO-seq (orange), or both. **(B)** Common DE genes in pairwise sequential transitions; for this comparison, LT (GRO-seq) and Ar (RNA-seq) are taken as comparable states. **(C–F)** Scatter plots of log2 fold change in RNA-seq vs. GRO-seq data for all 770 genes in the indicated pairwise transition (see panel **B**). DE genes for the indicated pair are plotted in dark red, all others are gray. Correlations for the 770 genes across each pairwise transition were: **(C)** IBA-SA, *r* = 0.76, *p*-value < 2.2e-16; **(D)** IBA-Ent, *r* = 0.204, *p*-value = 1.13e-08; **(E)** Ent-LT/Ar, *r* = 0.181, *p*-value = 3.97e-7; **(F)** IBA-LT/Ar, *r* = 0.174, *p*-value = 1.18e-06. **(G)** Top DE discordant protein coding genes in RNA-seq vs. GRO-seq data.

To gain a better understanding of the role of transcription in hibernation, we next compared the genes that were DE in both RNA-seq and GRO-seq datasets (Figure 6A). Merging the DE genes from the two datasets shows 770 were DE in both. Interestingly, 2350 DE genes from the RNA-seq dataset were not differentially transcribed and conversely, 1607 genes that were differentially transcribed by GRO-seq were not DE in the steady-state RNA population. We focused on the 770 genes that were DE in both the RNA-seq and GRO-seq datasets to examine their dynamics across the pairwise transitions. For this analysis, we considered the LT sample group in the GRO-seq dataset to be equivalent to the Ar group in the RNA-seq dataset.

While there were relatively few pairwise differences among these 770 genes common to both datasets (Figure 6B and Supplementary Table 5), their correlation plots were instructive (Figures 6C–F). Changes in transcription were well-correlated (*r* = 0.76) with changes in steady-state RNA in the SA to IBA comparison (Figure 6C), i.e., in the seasonal transition from homeothermy to heterothermy, even for the genes that did not meet our criteria for DE (*q* < 0.001) in both datasets. This result is consistent with a dominant role for transcriptional control in the seasonal reprogramming of liver gene expression during hibernation. In contrast, the correlations between the GRO-seq and RNA-seq datasets were far lower in the pairwise transitions across the torpor-arousal cycle among these 770 DE genes (*r* = 0.174-0.204). This finding suggests that the relative abundance of a transcript across the torpor-arousal cycle is more complex than a simple reflection of its transcription (Figures 6D–F). Although the dynamics of the five common DE genes between the GRO-seq and RNA-seq datasets in the Ent vs. Ar/LT were well correlated, the remaining 765 transcripts exhibited relatively little change in the steady-state RNA compared to their broad range of values for transcription across this pairwise transition (Figure 6E). Additionally, the pairwise comparison with the largest number of DE genes in both datasets, IBA compared to Ar in RNA-seq and LT in GRO-seq, revealed that the poor correlation between transcription and steady-state RNA across the torpor-arousal cycle (compare panel C in Figure 6 to panels D-F) reflected a conglomeration of positively and negatively correlated genes (Figure 6F). A mixture of positively and negatively correlated log2-fold changes was also apparent in the IBA-Ent comparison (Figure 6D and Supplementary Table 5) although far fewer of these were DE in both datasets.

As noted above, transcriptional control appeared to largely account for the observed changes in the transcriptome for the common DE genes between the homeothermic active animals and the hibernators (Figure 6C, illustrated by the gene, SCD_containing, in Supplementary Figure 6A) but not the relationship between transcription and the steady-state transcriptome in genes DE across the torpor-arousal cycle. Considering the 151 DE genes common to both datasets, 87 of them were positively correlated and can reasonably be considered to be transcriptionally controlled, either increasing (Supplementary Table 4, Arrdc3 in Supplementary Figure 6B) or decreasing in IBA. The remaining 64 negatively correlated DE genes between GRO-seq and RNA-seq either reached their highest steady-state RNA levels when transcription was lowest (Serpina7 and A2m) or conversely, had their lowest steady-state RNA levels when transcription was highest (Hsp90aa1 and Atp6v1b2, Figure 6G). Examining the GRO-seq coverage of these genes confirmed decreasing transcription from IBA to Ent and through the torpor bout with highest gene coverage in the GRO-seq data in IBA for Serpina7 and A2m. Conversely, increased GRO-seq coverage across Hsp90aa1 and Atp6v1b2 was apparent as animals transitioned from IBA to Ent and across the torpor bout (Supplementary Figures 6C–F).

We scanned the sequences of the positively and negatively correlated transcripts for enriched motifs in their 3′ non-coding regions. This analysis revealed a striking, reciprocal enrichment pattern for transcripts containing AU-rich motifs in the genes discordant between the RNA-seq and GRO-seq datasets (Supplementary Figures 7A,B). Specifically, steady-state transcripts that increased during IBA compared to Ar were enriched in AU-rich motifs when their transcription was decreased. Conversely, AU-rich motifs were depleted in those transcripts that were increased during Ar compared to IBA, despite their concomitantly low transcription. This result is consistent with the GC enrichment of transcripts that were stable across the torpor bout (Figure 2F) and implies regulation by RNA binding proteins that recognize AU-rich elements (García-Mauriño et al., 2017; Otsuka et al., 2019). Only five ARE-binding proteins were DE in the liver RNA-seq dataset, and, for all but one of these, the dynamics were different from hypothalamus (Supplementary Figure 7C). This difference is consistent with distinct regulation of ARE-containing transcript stability and turnover in hibernating liver compared to brain.

These liver RNA-seq data were further analyzed for qualitative changes associated with hibernation in addition to the quantitative changes described above. Specifically, we examined the liver RNA-seq data for deamination of adenosine by ADAR (adenosine deaminase acting on RNA), and for changes in splicing, both of which were found previously in hibernating brain (Riemondy et al., 2018; Fu et al., 2021).

### RNA Editing

**FIGURE 7:**
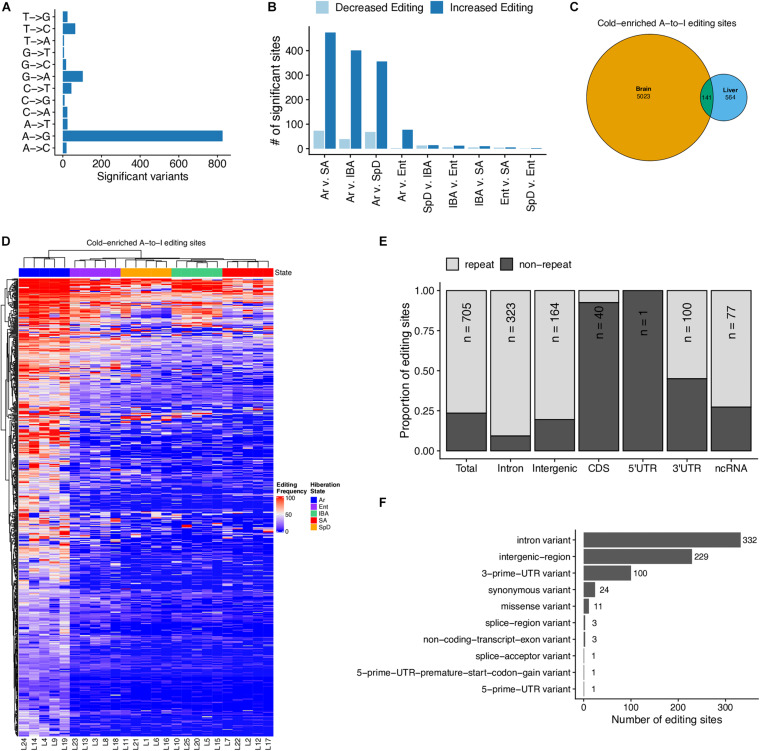
A-to-I RNA editing occurs in the liver during torpor. **(A)** Summary of single-nucleotide variants detected in RNA-seq data (but not in GRO-seq data) with allele frequencies that are significantly (FDR < 0.05) variable across hibernation stages. **(B)** Summary of pairwise analysis of A-to-I editing sites with differential editing frequencies between each stage. FDR < 0.05 is considered significant. **(C)** Euler-diagram comparing the A-to-I RNA editing sites with enhanced editing during torpor in brain tissue (Riemondy et al., 2018) and in liver, *p*-value < 2.3 × 10^– 308^. **(D)** Heatmap of the frequency of editing sites with cold-enriched editing in the liver. Only editing sites with sufficient counts in greater than six samples are shown. **(E)** Summary of the genomic positions of cold-enriched liver editing sites. **(F)** Summary of the predicted impacts of A-to-G substitution on mRNA processing and translational activities. See also Supplementary Table 6 and Supplementary Figure 8.

Our earlier study of the brain transcriptome in hibernating 13-lined ground squirrels revealed a large increase of adenosine deamination during torpor, i.e., when the animal’s Tb was low (Riemondy et al., 2018). This deamination effectively recodes RNA by converting adenosine to inosine, which behaves like guanosine. We asked whether a similar increase in edited sites was apparent in these liver RNA-seq data. As was observed in brain, a clear excess of A/G substitutions was observed in the RNA-seq data compared to all other possible variants (Figure 7A), and these are significantly elevated in Ar compared to all other pairwise comparisons, i.e., they accumulate at the low Tb of torpor in liver (Figures 7B,C). While there were fewer edited sites (705, Supplementary Table 6) in these liver data than were observed in brain, 141 sites were significantly edited in both tissues (Figure 7C and Supplementary Figure 8). And, in liver as in brain the edited sites were predicted to have little impact on the proteome, instead occurring largely outside of coding regions and in repeated DNA (Figures 7E,F).

### RNA Splicing

**FIGURE 8:**
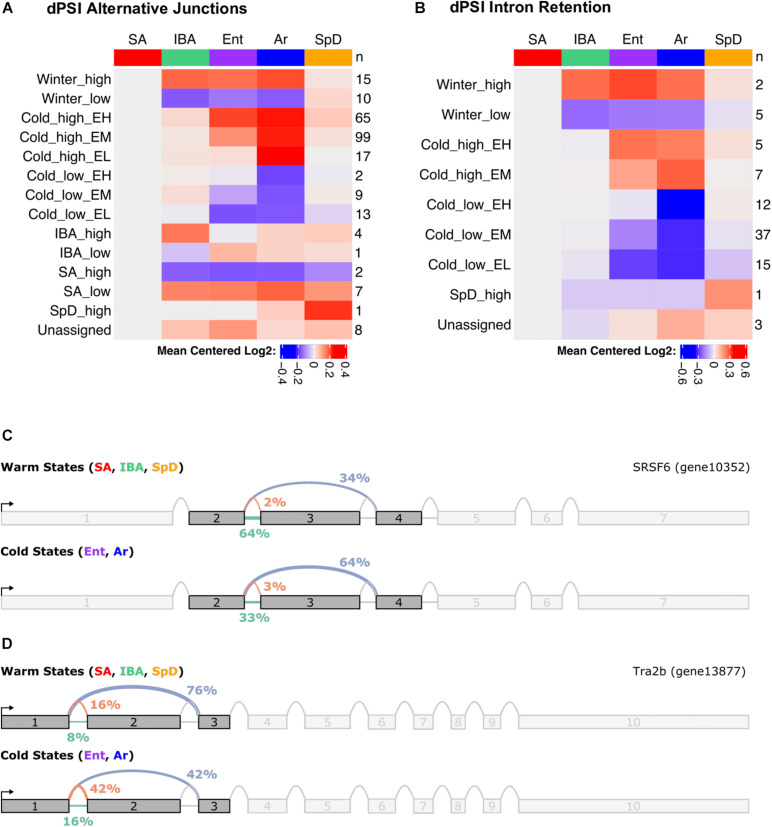
Summary of alternative splicing in the liver during hibernation. Heatmaps show summary of **(A)** mean dPSI, and **(B)** retained introns relative to SA. Numbers on the right give the number of genes in each of the indicated cluster patterns named on the left. Splice graphs from MAJIQ illustrate temperature dependent alternative splicing for **(C)** SRSF6 and **(D)** Trab2b. See also Supplementary Table 7.

Finally, the liver RNA-seq data were analyzed for alternative splicing during hibernation using MAJIQ (Vaquero-Garcia et al., 2016). MAJIQ reports alternative splicing events between states in terms of dPSI (change in “percent spliced in”) values for each possible junction that a given source (upstream) or target (downstream) sequence can form. MAJIQ refers to these collections of possible junctions as LSVs (local splicing variations). Here, we define alternative splicing as a significant increase in the PSI of all alternative junctions relative to the “summer-dominant junction” (most common junction in SA) at a given LSV. Consistent with our previous findings in the brain of the 13-lined ground squirrel (Fu et al., 2021), we found that alternative splicing in the liver is highly temperature dependent (Figure 8A and Supplementary Figure 9). As in the brain, we specifically observed that alternative splicing events most commonly exhibit the Cold_high_EM and Cold_high_EH patterns, indicating increased alternative junction use in the cold states (Ent and Ar) relative to the warm states (SA, IBA, SpD). Focusing on intron retention by considering the dPSI values of all retained introns rather than the most common junctions in SA, we observed that intron retention is largely restricted to Cold_low_EM and Cold_low_EL patterns (Figure 8B and Supplementary Figure 10). This finding is consistent with intron retention patterns in the brain, where intron retention is also the default heterothermic state for most LSVs that contain a significant state-specific intron retention event.

To determine if genes involved in certain pathways were consistently alternatively spliced in the liver during hibernation, we performed GO term enrichment on the list of genes with significant state-dependent alternative splicing events, independent of cluster (Supplementary Table 7). We found that the three most significantly enriched terms were “regulation of RNA splicing” (GO:0043484), “mRNA processing” (GO:0006397), and “RNA splicing” (GO:0008380). Strikingly, the top 14 most significant terms all concern RNA processing or RNA splicing, specifically. When considering the splicing clusters (Figure 8A) as separate gene lists (Supplementary Table 7), the same overrepresentation of splicing-related terms is returned, with the added information that most of the genes involved in splicing exhibit the Cold_high_EH pattern. This is consistent with our earlier findings that the splicing factors SRSF6 and Srsf5 are themselves alternatively spliced during hibernation in the brain and may directly modulate the state-dependent alternative splicing observed there (Fu et al., 2021). Indeed, we observe the same alternative splicing events in both Srsf5 and SRSF6 in the liver. Figures 8C,D show the splice graphs for two representative alternative splicing events in liver splicing factor mRNAs, SRSF6 and Tra2b.

## Discussion

Metabolic flexibility, the ability to maintain cell, tissue and organismal homeostasis despite variabilities in the internal and external milieux, is a critical characteristic of mammalian hibernation. Homeostasis is maintained despite the dramatic swings of nutrient intake, oxygen delivery and tissue temperature that occur during hibernation (Carey et al., 2003; Staples, 2016). We employed two high-throughput sequencing approaches to interrogate the steady-state transcriptome and transcription in the liver with the goal of revealing changes in liver gene expression that support the hibernating phenotype. RNA-seq quantifies the relative abundance of transcripts at steady-state, and therefore represents the product of transcription and RNA turnover (Mortazavi et al., 2008; Lovén et al., 2012). In contrast, GRO-seq quantifies pre-initiated RNA polymerases that are capable of continuing elongation when nuclei are incubated with nucleotides (Core et al., 2008). These datasets, obtained from precisely sampled livers representing key timepoints in both the torpor-arousal and seasonal cycles of hibernation, allowed us to separate both transcriptional from post-transcriptional and seasonal from temperature effects on liver gene expression in hibernation for the first time.

The central role played by the liver in the uptake and repackaging of dietary nutrients (Trefts et al., 2017), along with the demonstrated seasonal resistance of the 13-lined ground squirrel liver to damage caused by cold storage and reperfusion (Lindell et al., 2005) led us to expect a robust seasonal reprogramming of liver gene transcription and hence the transcriptome. Indeed, the dominant feature of the transcriptome was the seasonally decreased (Winter_low) abundance of a large number of transcripts for genes encoding metabolic enzymes, particularly those involved with fatty-acid, cholesterol and steroid biosynthesis, xenobiotic and amino acid metabolism including the urea cycle (Figure 3, Supplementary Figure 2, and Supplementary Table 2, 3). These enrichments are broadly consistent with gene enrichments found associated with short-term fasting in mice (Chi et al., 2020; Defour et al., 2020), and in previous analyses of the hibernating 13-lined ground squirrel’s liver metabolomic, transcriptomic and proteomic adjustments across hibernation cycles (Serkova et al., 2007; Hindle et al., 2014; Bogren et al., 2017), as well as in RNA-seq datasets of liver gene expression changes in other taxa of hibernators (Nespolo et al., 2018; Jansen et al., 2019). The basis for enhanced liver protection during hibernation is not quite as apparent in the changing transcriptome as the strong metabolic response (which may itself be protective, Hindle et al., 2014; Perez de Lara Rodriguez et al., 2017). But the seasonal gene enrichments in cell adhesion (Joutsen et al., 2020) and spermine metabolism (Pegg, 2014), both Winter_high, as well as enrichments in apoptotic cell clearance (Cold_high_EM), the unfolded protein response, regulation of cellular response to heat (Cold_low_EL), and hyaluronan metabolism (Day and de la Motte, 2005) in the torpor-arousal cycle may all contribute to protecting the functional integrity of the liver throughout hibernation.

Compared to our recent study of DE genes in three brain regions (Fu et al., 2021), this liver dataset is distinct in its elevated number of seasonally altered genes (44% in liver vs. 14, 20 and 23% in forebrain, hypothalamus and medulla, respectively), in the larger fold changes in liver (both a greater proportion of genes exhibiting > 2x fold-change, and the maximum fold changes observed, Supplementary Table 2) and in the large, liver specific enrichment of metabolic genes that are decreased over the entire winter hibernation season (Figure 3). This last feature in particular is consistent with earlier reports that the major component of differential gene expression in hibernation is related to tissue-specific gene expression (Grabek et al., 2015b; Bogren et al., 2017), and with the key role of the liver in maintaining metabolic homeostasis throughout the long winter fast, as discussed above. The generally larger fold-changes observed among the liver DE genes compared to the three brain regions likely reflects the relative enrichment of a single cell type, the hepatocyte, which comprises ∼80% of the cell volume in the mammalian liver. While the liver does have several other cell types and there is evidence of region-specific gene expression (Halpern et al., 2017; Trefts et al., 2017; Cheng et al., 2018) it is nonetheless not as heterogeneous as brain when considering the unique gene expression profiles of numerous small pools of neurons along with its numerous non-neuronal cell types. Regional functionalization in liver, taken together with our use of an ∼50-100mg broken piece of frozen liver for library preparations, may underlie the relatively high individual variation in the liver datasets, as evidenced by generally higher p-values (Supplementary Table 2), compared to the brain.

Many features of the liver RNA-seq dataset were shared with brain. RNA binding proteins, including polyA binding proteins, were increased for winter in liver (Supplementary Figure 2 and Supplementary Table 3), indicative of the importance of regulating RNA turnover and stability in the torpor-arousal cycle. The bias toward higher GC content among the genes stabilized across the torpor bout was also found in liver (Figure 2F). The preservation of this GC-rich subset of mRNAs may reflect the general depression of translation at the low Tb of torpor (van Breukelen and Martin, 2001) leading to decreased turnover of what would otherwise be highly translated, and consequently highly turned-over, mRNAs (Courel et al., 2019). The finding that several of the highest fold changes among the genes in liver that were DE across the torpor-arousal cycle were found in miRNA_containing genes was also a common feature with the three brain regions (Fu et al., 2021). In liver however, all of these dynamic miRNA_containing transcripts decreased dramatically across the torpor bout (i.e., between Ent and Ar, Figure 2E), and just one, MIRLET7D_containing, was also DE in brain. It is important to note that our library preparation methods do not capture mature miRNAs, so these quantifications do not address whether the functional miRNAs are DE. However, one of these, miR-148a, has been reported to be elevated during ET in hibernating 13-lined ground squirrel liver (Wu et al., 2016). Inspection of the read distributions for these miRNA_containing genes reveals that the majority of reads fall outside of the miRNA region, i.e., the quantification reflects the 3′ most remainder of the primary transcript excluding the miRNA (i.e., containing the polyA tail up to the 3′ end of the miRNA which has been largely processed out (Supplementary Figure 11). Additional experiments are needed to assess the functional significance of these dynamic miRNA_containing transcripts during torpor-arousal cycles, including whether the miRNA or the lncRNA that remains after initial processing (Du et al., 2019; He et al., 2021), or both, are functionally relevant.

The RNA subunit of RNaseP exhibited one of the largest increases seen in our RNA-seq dataset – log_2_-fold Ar/Ent = 5.95, Figure 2D. This large increase likely reflects novel polyadenylation of this gene during torpor, as demonstrated previously in brown adipose tissue (Grabek et al., 2015a). RNaseP RNA is not polyadenylated while serving its function in tRNA processing, and thus not normally captured in polyA selected RNA as used here for RNA-seq library construction. Rather the appearance of this transcript in our liver RNA-seq data (along with others, e.g., SCARNA17_like, Supplementary Table 2) likely indicates an uncoupling of the signal for and actual processing of transcripts through the nuclear exosome (LaCava et al., 2005; Yu and Kim, 2020) at the low Tb of torpor.

The direct impact of transcription on the differential-expression dynamics of thousands of genes in hibernation was assessed here for the first time. Our findings support and extend those of an earlier study in torpid and IBA hibernating golden-mantled ground squirrels compared to SA (van Breukelen and Martin, 2002) which used similar nuclear run-on techniques but was technically limited to global rather than gene-specific conclusions. As expected from those earlier results, our GRO-seq data revealed that a large number of liver genes contained pre-initiated RNA polymerase during early and late torpor and elevated transcription activity in IBA. Unexpectedly however, we found that transcription during torpor often extended 3′ of the transcription termination signal used in the warm Tb states, including into adjacent genes. Based on our new data and the literature we posit that transcription initiation and termination cease during torpor while elongation continues at a greatly reduced rate.

Several observations consistent with this hypothesis are: 1) run-off transcription measured globally using ^32^P-UTP incorporation is indistinguishable between ET and LT, reflecting the maintenance of a constant number of elongation-competent RNA polymerases for multiple days at 4-8°C across the torpor bout (van Breukelen and Martin, 2002), because the energetic requirements of the three states of transcription (initiation, elongation and termination) are quite different (Yan and Gralla, 1997; Han et al., 2016) the elongation-competent polymerases present in ET likely remain present in LT; 2) browser tracks of genes with significantly increased transcription only during torpor revealed read-through transcription from upstream genes and lacked evidence of local promoter-proximal pausing as expected if initiation had occurred on their own promoters (Figures 4A,B and Supplementary Figures 3B–E), moreover, these genes exhibited no increase of steady-state RNA across the torpor bout, and they are typically not expressed in liver in mouse or human, consistent with their transcription coming from an upstream promoter and a lack of termination on that upstream gene; 3) while the GRO-seq data revealed no difference in the distribution of polymerases surrounding the TTS between SA and IBA, we observed an increasingly downstream shift as animals progressed into and through the torpor bout, i.e., from IBA - > Ent - > ET - > LT (Figure 4C and Supplementary Figure 3G), again consistent with the movement of elongating polymerase and lack of termination; and 4) despite dramatic, temperature-dependent effects on the rate of transcriptional elongation in run-off assays, a small amount of incorporation is detectable at the temperature of a torpid hibernating ground squirrel (van Breukelen and Martin, 2002), indicating that elongation can proceed at the temperature of torpor. In the absence of *de novo* initiation or termination of transcription during torpor, the difference between the mean location of polymerase downstream of TTSs in ET and LT can be taken to reflect the movement of elongating RNA polymerase across the torpor bout. Specifically, in these samples RNA polymerase moved 1890 ± 29 nt 3′ from ET to LT (calculated as the difference between mean ET and LT fstitch extensions) in 7.3 ± 2.2 days (the difference in mean times with Tb below 8° for the ET and LT animals). Thus, the elongation rate observed across the torpor bout was ∼ 2kb in 5 days, or about 0.0003 kb/min, a rate far lower than the typical PolII transcription rate of 1-6 kb/min (Fuchs et al., 2014; Veloso et al., 2014). Additional experiments such as pulse-chase assays (Fuchs et al., 2014) will be necessary to estimate the elongation rate more precisely during torpor.

The increased read-through transcripts detected in the GRO-seq data during torpor typically did not lead to increased steady-state abundance of their corresponding transcript (i.e., they were not DE in the RNA-seq data). Therefore, normal 3′ end cleavage and polyadenylation is apparently greatly suppressed during torpor. Significantly, a set of recent papers report that several stress conditions, including heat shock, hypoxic and osmotic stress, as well as viral infection, cause suppression of transcriptional termination leading to read-through transcription in numerous genes (Vilborg et al., 2015, 2017; Vihervaara et al., 2017; Cardiello et al., 2018). These findings are strikingly similar to what we see occurring naturally in the liver of hibernating ground squirrels across the torpor bout with the important distinction that there is no evidence to suggest whether proper transcripts can be processed from those read-through transcripts when the stress stimulus is reversed. Read through transcription has also been shown to alter chromatin architecture (Heinz et al., 2018) and hence which genes are readily accessible to activation by transcription factors. Significantly, Hsf1, which we found to be induced throughout winter hibernation (Winter_low, Supplementary Table 2; squirrelBox), has been linked to altered transcriptional response and chromatin architecture genome wide (Vihervaara et al., 2017; Vilborg et al., 2017).

One finding from the earlier study of transcription during hibernation was not apparent in our GRO-seq data; liver transcription in golden-mantled ground squirrels increased in IBA compared to both LT and SA, with IBA approximately 2-fold higher than LT and 1.3-fold higher than SA (van Breukelen and Martin, 2002). In contrast, among the 2417 DE genes in our GRO-seq data, there were approximately equal numbers of highly transcribed genes during SA and IBA, and slightly more in LT (Supplementary Figure 5), although the LT genes were not as strongly transcribed (Figure 5C). This is likely explained by the library-size based normalization that was applied to the RNA and GRO-seq data. This normalization assumes that RNA yield is similar across cells in each sample, which will therefore obscure changes in overall transcriptional activity between hibernation stages (Lovén et al., 2012). This data-processing step, along with higher rates of transcription in IBA and SA and the large number of genes that were excluded in our analyses with its focus on the differentially transcribed genes would all contribute to this apparent discrepancy.

Another striking and noteworthy feature of the GRO-seq data is that for most genes, whether the gene will exhibit increased or decreased polymerase during torpor is well-correlated with whether polymerase during Ent on that gene was increasing (Cold_high_EM) or decreasing (Cold_low_EM). This feature of transcription in the torpor-arousal cycle was most readily apparent in the heatmap in Supplementary Figure 5; genes in Cold_high_EM and IBA_low_EM have increased coverage in GRO-seq from IBA to Ent, whereas genes in Cold_low_EM and IBA_low_EM were decreasing from IBA to Ent. This pattern suggests that the bulk of transcriptional control across the torpor bout has been established prior to Ent.

Seasonal changes in liver transcripts were well-correlated with their transcriptional dynamics (Figure 6C and Supplementary Figure 6A), as were many of the transcripts DE across the torpor-arousal cycle (positively correlated in Figures 6D–F and Supplementary Table 5). The dynamics of these positively correlated transcripts thus likely reflects their differential transcription.

The regulation of the negatively correlated genes across the torpor-arousal cycle is more complex, however. While we cannot conclusively rule-out transcriptional control during torpor, most transcripts that accumulate during torpor appear to be unusually stable rather than newly transcribed. This conclusion is based on a common pattern of low GRO-seq coverage in ET and LT for genes with high relative abundance in the RNA-seq data (upper left quadrant in Figure 6F). This pattern is exemplified by the genes encoding two plasma proteins secreted from liver, SerpinA7 and A2M (Figure 6G and Supplementary Figures 6C,D). SerpinA7, formerly known as TBG (thyroxine binding globulin), is a major carrier of thyroid hormone and A2m, alpha-2-macroglobulin, is a protease inhibitor and cytokine transporter which can inhibit inflammatory cascades (Sieckmann et al., 2014); both were previously shown to be elevated in hibernating ground squirrels (Srere et al., 1992; Epperson and Martin, 2002; Hindle et al., 2014). Transcripts of genes with the opposite pattern, i.e., elevated in the GRO-seq data during torpor when they are at their lowest relative abundance in the RNA-seq data often reach their highest level during IBA (lower right quadrant in Figure 6F). This pattern is exemplified by Hsp90aa1 and Atp6v1b2 (Figure 6G and Supplementary Figures 6E,F). Their high GRO-seq coverage in ET and LT suggests that additional RNA polymerases are still able to initiate and escape into elongation mode after the Ent but prior to ET sampling timepoints, although we found no evidence of further increased transcription between ET and LT, and there was no corresponding increase in the steady-state RNA (RNA-seq) between Ent and Ar for these genes. Rather, their increased mRNA abundance in IBA in the absence of transcription (GRO-seq low in IBA) suggests that the transcripts initiated as the animals entered torpor were slowly elongated throughout the torpor bout, but not processed by cleavage and polyadenylation until the animal rewarmed, i.e., early in IBA. While the aforementioned transcripts that were stabilized across the torpor bout would be first translated when Tb recovers during arousal (van Breukelen and Martin, 2001), the next set of sequentially expressed genes would be those already largely or completely transcribed during torpor after termination and release. Such a temporal pattern could set up a repeating, cyclical gene expression pattern that specifies all of the necessary components of the torpor-arousal cycle including recovery of functional integrity, restoration of metabolic homeostasis and preparation of the next bout of torpor.

Along with the quantitative dynamics of the transcriptome discussed above, analysis of these RNA-seq data also revealed qualitative changes related to hibernation. Deamination of adenosine by ADAR, picked up in our sequencing as A-to-G transitions, increased across the torpor bout (Figure 7 and Supplementary Figure 8). As described previously for the brain transcriptome (Riemondy et al., 2018), the vast majority of these occurred outside of coding sequences, consistent with a role for them in preventing activation of innate immunity through the double-stranded RNA sensing pathway (Liddicoat et al., 2015). It is interesting to speculate an increased need for protection against double-strand RNA given the increased transcription of intergenic regions during torpor that was revealed by GRO-seq analysis.

We observed functional enrichment of terms related to mRNA splicing in gene clusters linked to both the seasonal (Winter_high) and torpor-arousal cycles in RNA-seq data (Cold_high and Cold_low _EM, Supplementary Table 3) and in the list of genes with altered splicing (Supplementary Table 7). Consistent with these enrichments, multiple genes exhibited differential splicing, with the bulk of changes occurring in the torpor arousal-cycle, including in splicing factors. The implications of the SRSF6 alternative splicing event are discussed in detail in our previous analysis of the brain transcriptome (Fu et al., 2021). Interestingly, the observed splice variation in Tra2b (Transformer-2 protein homolog beta) is also likely to alter splicing, possibly in concert with SRSF6. Trab2b encodes a highly conserved sequence-specific RNA-binding protein that both regulates mRNA splicing and binds directly to SRSF6 among other proteins (Hegele et al., 2012). Alternative splicing of the human TRA2B mRNA produces several protein isoforms, including a 288 amino acid “complete” isoform (ENST00000453386) and a 188 amino acid truncated form (ENST00000382191) caused by skipping the second exon which lacks the first of two RS (Arg/Ser-rich) domains. In humans, there is evidence that the full-length protein is responsible for the role of TRA2B in alternative splicing, and that TRA2B is involved in alternative splicing of its own mRNA (Hofmann et al., 2000; Stoilov et al., 2004). In the 13-lined ground squirrel, the two most common mRNA isoforms produce highly conserved 288 amino acid and 188 amino acid proteins that correspond directly to the two most common human isoforms. Incredibly, however, the squirrel generates the conserved short protein isoform through the *inclusion* of a second exon (and resulting frameshift) rather than exclusion of an exon, as in the human gene. In liver, the long protein isoform (exclusion of exon 2) was dominant in warm states, while the short protein isoform (inclusion of exon 2) increased in the cold (Figure 8C). As the Tra2b long protein isoform is reported to be required to regulate alternative splicing in humans, this pattern appears to be functionally inverted relative to SRSF6 (Fu et al., 2021), indicative of a complex interaction between splicing factors during hibernation.

## Conclusion

In conclusion, these liver data demonstrate the necessity and value of carefully timed samples when studying differential gene expression associated with the dramatic physiological transitions of hibernation. These findings provide both a resource and a roadmap to guide future work that exploits the physiological dynamics of hibernation to discover the underlying molecular mechanisms of metabolic suppression and tissue protection. While we chose to focus our analysis and discussion of these data on more general features of transcriptional and post-transcriptional gene regulation, these lists of differentially expressed genes provide a rich resource to guide future hypothesis-driven work and squirrelBox offers ready access and analytical tools for exploration of these data for individual genes of interest. The secrets that enable the hibernator’s unique ability to withstand physiological extremes lethal to non-hibernating mammals, including humans, is expected to lie among these differentially expressed genes. Such understanding will be foundational to the ability to safely engineer reversible metabolic depression in non-hibernators, including humans.

## Materials and Methods

### Banked Liver Tissue

Liver was dissected from animals in various physiological states across their circannual rhythm based on calendar date and Tb measured using implanted telemeter or ibutton, or rectal probe, flash frozen in liquid nitrogen and then stored below -70°C continuously until use, as described (Hindle et al., 2014). Five states were used for RNA-seq: SA, summer active (8 Aug); IBA, interbout aroused (11 Feb–26 Mar, 2–3 h after Tb reached 30°C following > 5d torpid with Tb < 6.5°C, with Tb 30.6-37.7°C); Ent, entrance into torpor (3 Jan–31 Jan, 23–27°C after 10-14 hr with Tb > 30°C); Ar, arousing from torpor (28 Dec–22 Jan, with Tb spontaneously recovered to 7.9-12.8°C after multiple days < 6.5°C); and SpD, spring dark (21 Mar–30 Apr, after at least 10 consecutive days with Tb 35.5-37°C). For GRO-seq, we used the first three of the above states (SA, IBA, and Ent), plus ET (3 Dec–13 Jan with Tb < 30.0°C following an IBA for 8-16% of the previous torpor bout duration) and LT (31 Jan–22 Feb, 5.5–5.7°C for 80-95% of the previous torpor bout duration). The animal metadata are provided in Supplementary Table 1.

### RNA-Seq

50-100mg of frozen liver were pulverized under liquid nitrogen and then homogenized in ice cold TRIzol using a polytron (Brinkman). RNA was further purified from the aqueous phase using a Direct-zol RNA MiniPrep Kit (Zymo Research). RNA was assessed for quantity (NanoDrop, Thermo Scientific) and quality (average RIN values were 8.3 ± 0.4 with no differences among sample groups; Bioanalyzer, Agilent Technologies, Supplementary Table 1). 1 μg was submitted to Genewiz, Inc. (Plainfield, NJ), for library preparation and sequencing. Strand-specific RNA-seq libraries were constructed using the TruSeq Stranded mRNA Library Prep Kit (Illumina). Paired-end sequence reads (150nt) were collected on an Illumina HiSeq4000 and demultiplexed fastq files were returned to Colorado for analysis.

### RNA-Seq Data Processing

The raw sequencing reads were first trimmed with cutadapt to remove adaptor sequences, short sequences (-m17) and low-quality bases (-q10, Martin, 2011). These sequences have been submitted to GEO (accession number GSE166814). Trimmed reads were mapped to the mitochondrial genome with hisat2 (Pertea et al., 2016, Supplementary Table 1). The read distribution across genes for each sample was assessed using the geneBody_coverage function in RSeQC (v4.0.0); this analysis revealed an elevated 3′ bias in a few samples that were distributed across the sample groups (Supplementary Figure 12A). Non-mitochondrial reads (92.2 ± 2.3%) were mapped to transcript annotations on the HiC_Itri_2 genome (Fu et al., 2021) using Salmon (Patro et al., 2017) with –numBootstraps 50 (Supplementary Table 1). Salmon assignments were imported into R (R Core Team, 2019) and summarized by gene using tximport (Soneson et al., 2015). Only genes with rlog ≥ 7 in at least 4/5 individuals from at least one group were retained for further analyses. These values (Supplementary Table 2, Supplementary Data Sheet 3) were used as input for random forests (Breiman, 2001), plotting, WGCNA (Langfelder and Horvath, 2008) and clustering algorithms. Differentially expressed genes (DE genes) were defined using DESeq2 (Love et al., 2014) as those with a likelihood ratio test (LRT) adjusted p-value of ≤ 0.001 across all states. The model included a term to control for the effect of sex. DESeq2 was also used to calculate “shrunken” log2 fold-changes (Stephens, 2017) for differentially expressed genes and normalized, transformed (rlog) count matrices (Supplementary Table 2). Comparison of sample cluster patterns by random forest documents that hibernation physiology explains a large fraction of the variance across the data among these samples (Supplementary Figures 12B–D).

### Cluster Analyses

WGCNA: Pseudocounts were processed with ComBat (Johnson et al., 2006) to remove effects of sex and then passed to WGCNA (v1.68, Langfelder and Horvath, 2008) for module detection. Parameters were optimized to construct a signed network (TOMType = “signed”, networkType = “signed”), high sensitivity (deepSplit = 3), with more aggressive than default merging and reassignment (mergeCutHeight = 0.25, reassignThreshold = 1). Additional settings were: minModuleSize = 30, minCoreKME = 0.5, minKMEtoStay = 0.4. Genes from each module were inspected in comparison to their summary profile eigengene. Module–trait associations quantification was used to identify modules that were significantly associated with the measured traits and their correlation values were color coded for plotting.

Reference pattern clustering: Gene expression patterns were assigned to their final reference clusters by calculating Pearson correlation coefficients. Mean expression values were calculated for each gene in each state, and these average expression values were tested for correlation with the 14 most common patterns informed by the WGCNA modules. Genes (r ≥ 0.8) were assigned to the cluster where most highly correlated; no ties were observed. Genes with *r* < 0.8 for any reference cluster were defined as Unassigned.

### Gene Enrichment Analysis

Lists of the clean_gene_symbol (Supplementary Table 2) for genes in each cluster (RNA-seq) or common (GRO-seq and RNA-seq) pattern were submitted to DAVID (Huang et al., 2009) for gene enrichment analysis. Gene symbols were mapped to human and analyzed using the chart option with the human gene set as background. GO terms for all *p* Value < 0.01 were extracted and the biological process (BP) terms were further analyzed and visualized using REViGO (Supek et al., 2011) at 0.9 “allowed similarity” between terms.

### Global Run On Sequencing (GRO-Seq)

The nuclear run-on assay was performed and analyzed as described previously (Fang et al., 2014). Nuclei were extracted from frozen ground squirrel liver that had been stored continuously at < -70°C after immediate freezing in liquid nitrogen. Approximately 50mg of liver were homogenized (Dounce) in ice-cold lysis buffer. Aliquots of nuclei preparations were inspected visually for quality and counted using a Countess Automated Cell Counter (Invitrogen). The nuclear run-on reaction was performed on ∼40 million nuclei for 7min. All GRO-seq library preparations were done in parallel to avoid batch effects. Libraries were sequenced on an Illumina HiSeq2000.

### GRO-Seq Data Processing

The raw sequencing reads were trimmed with serial runs through cutadapt to remove the adaptor sequence and polyA, short sequences with settings -z, -m32 (Martin, 2011). These sequences have been submitted to GEO (accession number GSE166370). Trimmed reads were mapped to the HiC_Itri_2 genome (Fu et al., 2021) using BWA and visualized using IGV and UCSC genome browsers. Reads were enumerated across gene regions in each sample using the read_distribution function of RSeQC (Supplementary Figure 13A). The reads were also independently mapped using a splice-aware aligner (STAR v 2.5.2a) to assess the level of mRNA contamination which was found to be low and minor across all libraries (Supplementary Figure 13B). Uniquely aligned (BWA) reads overlapping genes were quantified using featureCounts from the subread package (Liao et al., 2014). Representative gene coordinates were generated by merging overlapping coordinates of each transcript for each gene. Differentially transcribed genes were identified using DESeq2 (Love et al., 2014), initially using the HiC_Itri2 annotations used for RNA-seq. In the second analysis, existing transcript annotations were modified by building fstitch (Azofeifa et al., 2017) annotations, first for each state using the merged triplicate reads. These were then merged across all states to generate maximal called transcript regions which were intersected with the HiC_Itri_2 annotations. In cases where the fstitch annotation extended further 3′, roughly half the genes (Supplementary Table 4), the fstitch annotation was substituted for that gene’s annotation, otherwise the annotation was unchanged. All overlapping genes were then merged, with the gene name retaining all merged gene names separated by a “:”. These fstitch gene annotations were further processed to exclude the first 500nt (based on TSS annotation, Vihervaara et al., 2017). Read counts were re-quantified on this new annotation using featureCounts. DE genes among the GRO-seq reads mapping to each of these unique gene bodies were identified using DESeq2 and further filtered to exclude gene bodies overlapping multiple genes or annotations < = 50 nt in length (Love et al., 2014).

Metagenes of GRO-seq read coverage were constructed by defining genomic intervals -1 and + 5kb (TTS) or -1 and + 1kb (TSS) centered on gene end coordinates. Genes with overlapping coordinates after extension were excluded to limit analysis to well-separated, non-overlapping genes. Additionally, genes containing gap regions of undefined sequence (i.e., > N_20_) in these regions were also discarded. Library-sized normalized counts were averaged across 50bp (TTS) or 10bp (TSS) bins, then scaled by the maximal expression within each metagene interval, followed by averaging values in each bin across all genes. Well-expressed genes were selected for metagene analysis, requiring GRO-seq expression of at least 25 TPM in either SA or IBA, based on full gene coordinates, with coverage in at least 33% of the bins in 3 or more samples. Characteristically, several features of the GRO-seq data were distinct from the RNA-seq data, most notably the read coverage of introns was greatly enhanced in the GRO-seq data (Supplementary Figure 13A). In addition, few GRO-seq reads were purely exonic (Supplementary Figure 13B) and, while greater GRO-seq sequencing depth as well as improved genome contiguity and annotation would improve their detection, many genes provide evidence of promoter proximal pausing and divergent initiation as first revealed by GRO-seq (Core et al., 2008).

### Sequence/Motif Analysis

Sequence analyses were carried out in R, utilizing a forged BSgenome data package from the newly built genome assembly (Fu et al., 2021), which enables quick queries of untranslated sequences. In addition, k-mer counting and enrichment statistical analysis are achieved through the R package transite (Krismer et al., 2020).

### RNA Editing

The RNA-seq and GRO-seq data were processed as described previously (Riemondy et al., 2018) to identify variant sites except these were mapped to the new HiC_Itri_2 genome (Fu et al., 2021). As expected there were few state-dependent A-to-G variant sites in the GRO-seq data and none were enhanced in the cold. But 14 of the 719 A-to-G sites with cold enriched editing in the RNA-seq data were found in the list of variants detected in the GRO-seq data. Because these are likely polymorphisms, they were excluded from further analyses. The remaining 705 A/G variants in the RNA-seq data with FDR < 0.05 were considered ADAR edited sites. SnpEff was used to predict the functional impact of A-to-I editing (Cingolani et al., 2012).

### Alternative Splicing Analysis

All reads with at least 20 nt after trimming were aligned to the HiC_Itri_2 genome using STAR (Dobin et al., 2012) in two-pass mode with: –limitSjdbInsertNsj 2000000, –outSAMattributes NH HI AS nM MD, –alignSJoverhangMin 8 on the second pass. Splice graphs were assembled from the STAR alignments using MAJIQ Builder and changes in relative local splice variation (LSV) abundance (delta PSI = dPSI) between states were identified using MAJIQ Quantifier (Vaquero-Garcia et al., 2016). Significant LSVs, defined as a 99.9% probability of a dPSI of at least 0.2 in any pairwise state comparison, were visualized by separately plotting the abundance of the SA-dominant splice junction and retained intron (if present) relative to SA using ComplexHeatmap (Gu et al., 2016) in R. Reference pattern clustering was done as described above. Protein domains in alternative splicing isoforms were identified using HMMER version 3.3^1^ on version 32.0 of the PFAM database (El-Gebali et al., 2018).

### Data Exploration

RNA-seq and GRO-seq analyses described above are integrated with the genome assembly and transcriptome annotation (Fu et al., 2021) into an interactive R Shiny browser, squirrelBox, which is hosted online https://raysinensis.shinyapps.io/squirrelbox_liver/or can be downloaded and run locally https://github.com/rnabioco/squirrelbox. squirrelBox enables data filtering, query, high quality plotting, and basic GO-term and kmer enrichment analyses. Gene set distribution along the HiC_Itri_2 genome (RefSeq GCF_016881025.1) is powered by genomic interval manipulation via valr (Riemondy et al., 2017) and JavaScript-interfacing via Biocircos (Cui et al., 2016). squirrelBox was originally designed to explore brain RNA-seq data (Fu et al., 2021) but here we demonstrate its flexibility to input datasets and ready adaptation to other data exploration needs.

### smORF Annotation in squirrelBox

Sequences from all annotated transcripts were processed via the micropeptide prediction tool MiPepid. Reported short open reading frames of length ≥ 10 aa, in the MiPepid classification class of “coding”, and probability ≥ 0.9 were labeled as “MiPepid_predicted”. The similarities of these sequences to high confidence smORFs documented in the SmProt database were then calculated using blastp-short parameters for sequences of length ≤ 30 aa and default parameters on longer sequences. Homologous smORFs were annotated with the SmProt micropeptide when blastp results escore ≤ e-10, percentage identical ≥ 90, and alignment coverage of the subject sequence ≥ 0.9. These analyses are provided as columns in the squirrelBox gene summary table for exploration.

## Data Availability Statement

The datasets presented in this study can be found in online repositories. The names of the repository/repositories and accession number(s) can be found in the article.

## Ethics Statement

The animal study was reviewed and approved by the University of Colorado School of Medicine Institutional Animal Care and Use Committee (protocol #44309).

## Author Contributions

AG, RF, and KR analyzed data, and drafted and reviewed manuscript. JJ prepared GRO-seq libraries and reviewed manuscript. BF analyzed data and reviewed manuscript. ML designed study and reviewed manuscript. SM designed study, prepared RNA, analyzed data, and drafted and reviewed manuscript. All authors contributed to the article and approved the submitted version.

## Conflict of Interest

The authors declare that the research was conducted in the absence of any commercial or financial relationships that could be construed as a potential conflict of interest.

## Footnotes

1 hmmer.org
