# Supplementary material for: Liver Transcriptome Dynamics During Hibernation Are Shaped by a Shifting Balance Between Transcription and RNA Stability
Source: Front Physiol. 2021 May 21;12:662132. doi: 10.3389/fphys.2021.662132 (PMC8176218; doi:10.3389/fphys.2021.662132)
Supplement: Supplementary file 1 [file Data_Sheet_1.PDF]

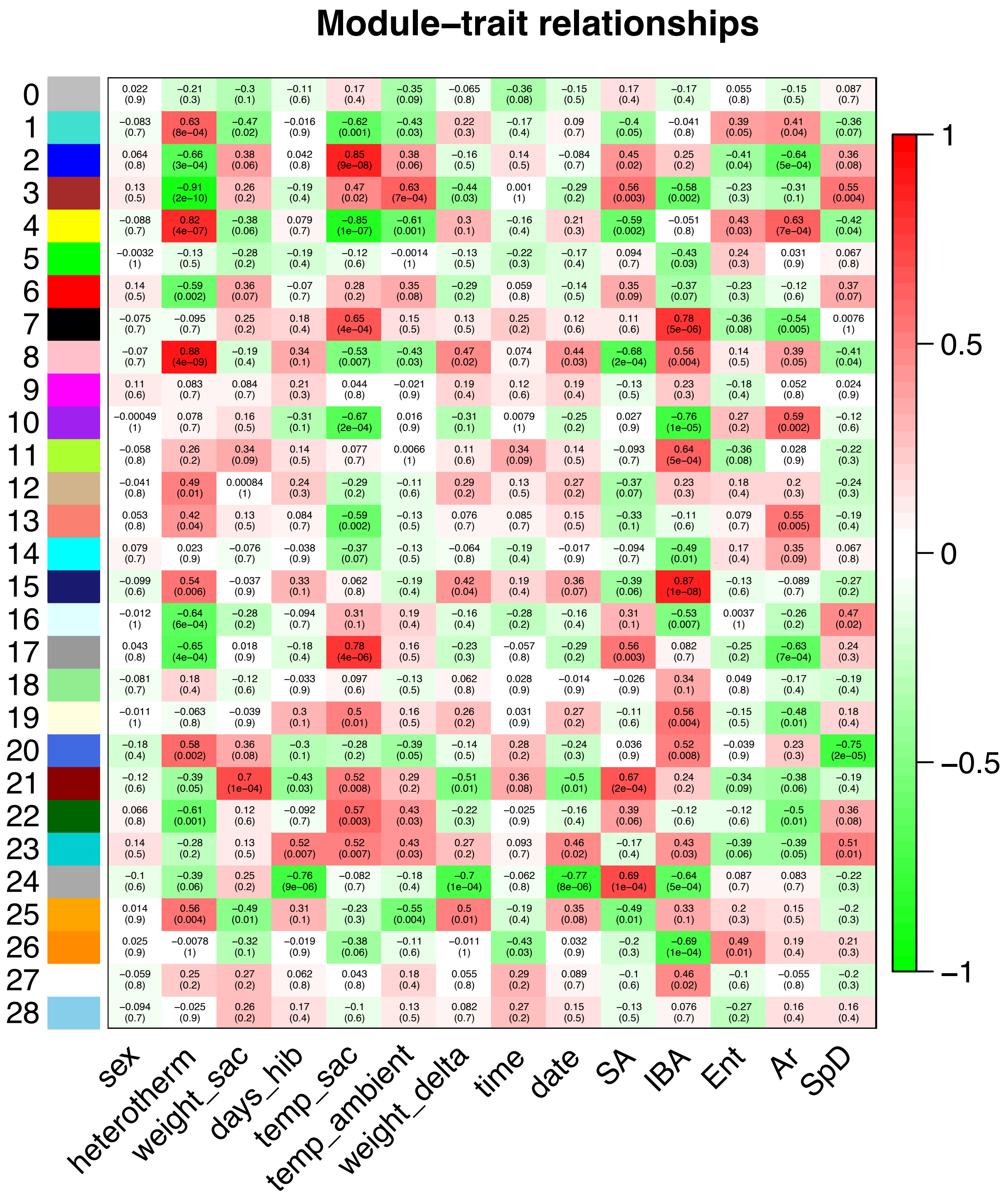

Supplementary Figure 1. WGCNA RNA-seq cluster-phenotype correlations in liver. Colors (n=28) on the left represent clusters of co-expressed genes. Numbers in each box report the correlation (top number) and its significance (bottom number, in parenthesis) between each cluster and the phenotypes listed across the bottom. Tested phenotypes were based on the information in Supplementary Material, Supplementary Table 1.

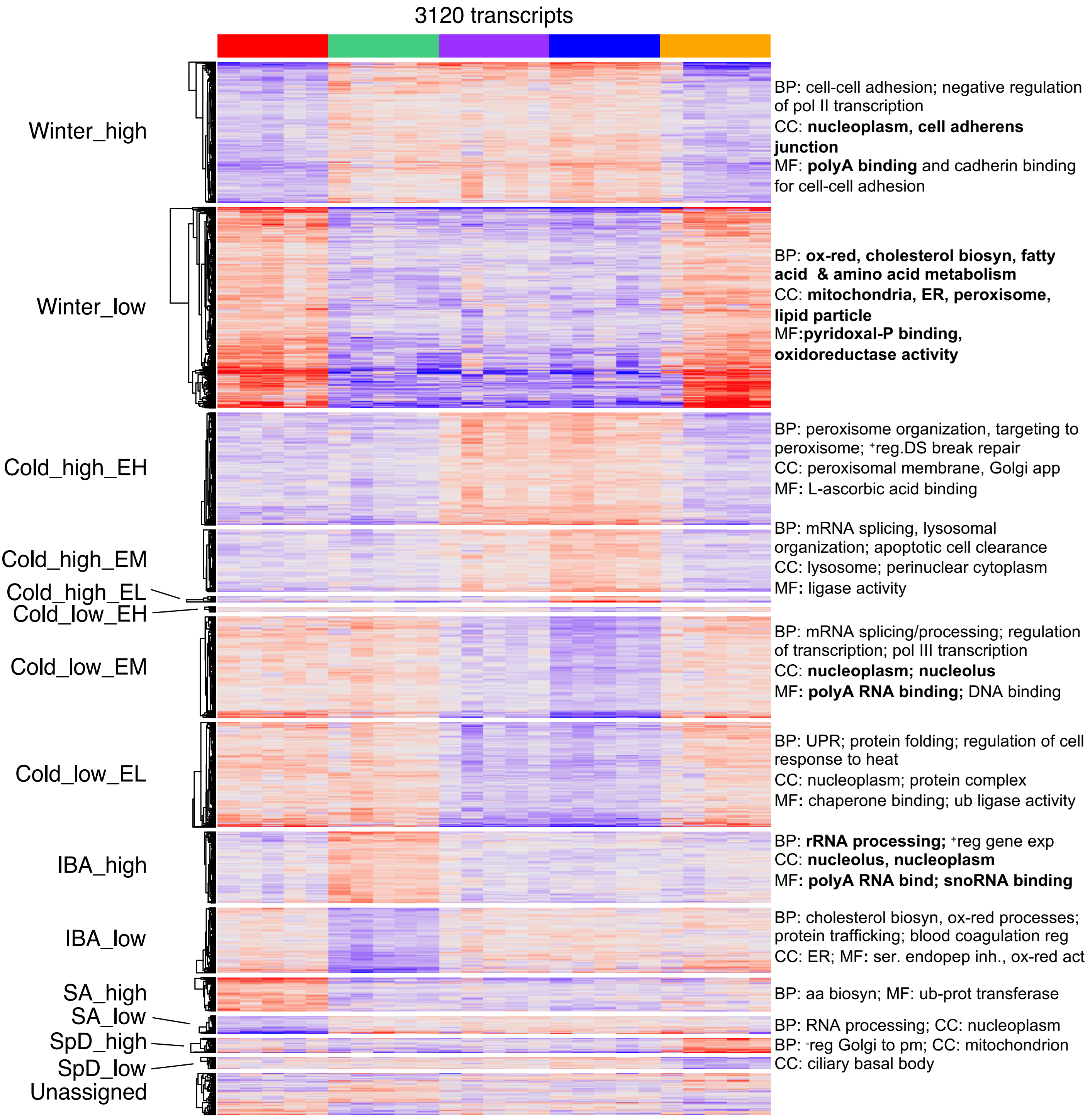

Supplementary Figure 2. Heatmap of DE genes by RNA-seq, clustered by expression pattern. Top gene enrichments for each cluster are indicated on the right for GO terms, Biological Process (BP), Cell Component (CC) and Molecular Function (MF). Abbreviations used are: act, activity; aa, amino acid; app, apparatus; bind, binding; biosyn, biosynthesis; ER, endoplasmic reticulum; exp, expression; inh, inhibitor; ox-red, oxidation-reduction; pm, plasma membrane; prot, protein; $^{-}$ reg, $^{+}$ reg, reg, negative, positive or just regulation, respectively; pser, serine; ub, ubiquitin; UPR, unfolded protein response. See also Supplementary Table 3.

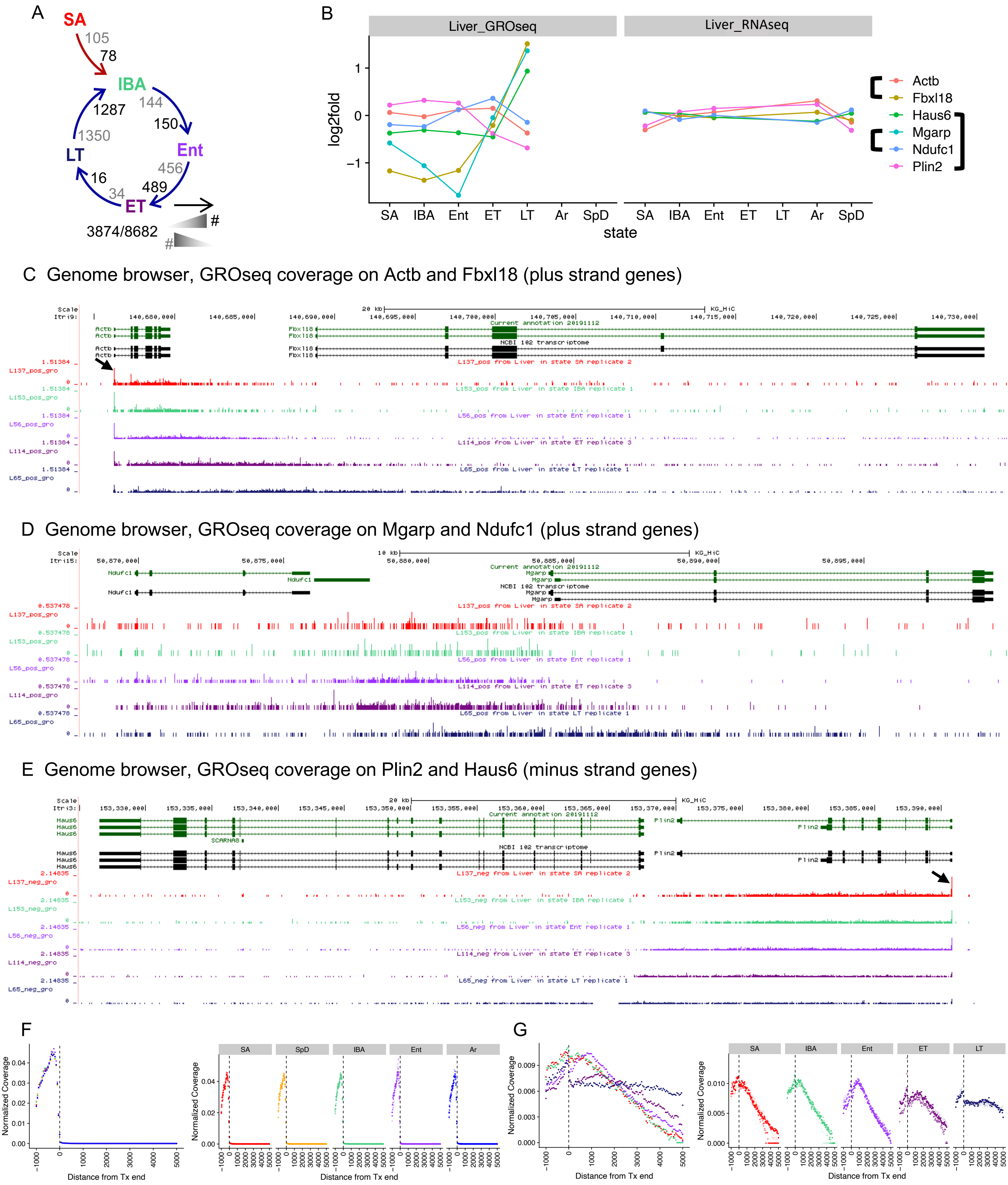
A
SA
105
78
IBA
144
150
Ent
456
489
ET
34
16
LT
1350
1287
3874/8682
#
#
B
Liver_GROseq
Liver_RNAseq
log2fold
1
0
−1
SA IBA Ent ET LT Ar SpD
SA IBA Ent ET LT Ar SpD
state
Actb
Fbxl18
Haus6
Mgarp
Ndufc1
Plin2
C Genome browser, GROseq coverage on Actb and Fbxl18 (plus strand genes)
L137_pos_gro
L153_pos_gro
L56_pos_gro
L114_pos_gro
L65_pos_gro
D Genome browser, GROseq coverage on Mgarp and Ndufc1 (plus strand genes)
L137_pos_gro
L153_pos_gro
L56_pos_gro
L114_pos_gro
L65_pos_gro
E Genome browser, GROseq coverage on Plin2 and Haus6 (minus strand genes)
L137_neg_gro
L153_neg_gro
L56_neg_gro
L114_neg_gro
L65_neg_gro
F
Normalized Coverage
Distance from Tx end
SA
SpD
IBA
Ent
Ar
G
Normalized Coverage
Distance from Tx end
SA
IBA
Ent
ET
LT

Supplementary Figure 3. Initial assessment of differential transcription during hibernation by GRO-seq. A) Schematic of pairwise transitions; the fall seasonal change from homeothermy to heterothermy is represented by (SA -> IBA) and, within heterothermy, the torpor-arousal cycle by (IBA -> Ent -> ET -> LT); the numbers of DE genes defined by DESeq2 analysis of GRO-seq data, increased or decreased, across each pair are given by the black and gray numbers, respectively, as indicated in the schematic below. Numbers below are the number of DE genes/total pass filter genes evaluated. B) Linegraphs plot pairwise log2 fold changes for six genes in GRO-seq and RNA-seq data. The three genes increased in GRO-seq between ET and LT were all downstream of a transcriptionally active but not DE gene (left panel). None of these six genes were DE by RNA-seq (right panel, brackets indicate adjacent gene pairs). C-E) Genome browser views of genomic region with GRO-seq coverage in representative sample from each state for C) Actb and Fbx18l, D) Ndufc1 and Mgarp, and E) Plin2 and Haus6. F, G) metagene plots show average normalized coverage near transcriptional termination signal for the same 635 genes (see Methods) in: F) RNA-seq or G) GRO-seq data. Left panels in each pair plot average of n=5 samples for RNA-seq, n=3 samples for GRO-seq (except GRO-seq LT, n=2) for each state across all genes, right panels plot individual samples for all genes.

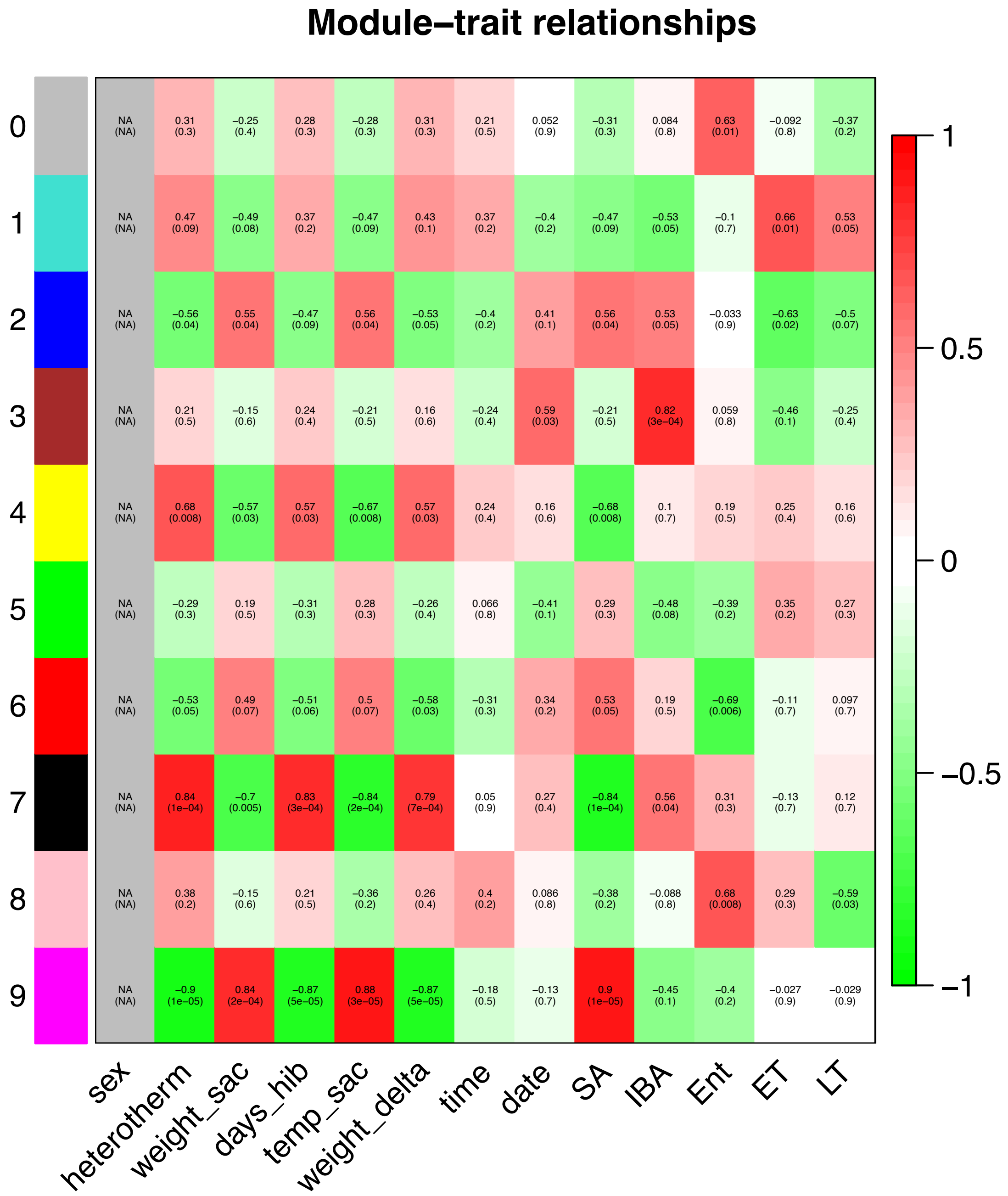

Supplementary Figure 4. WGCNA GRO-seq cluster-phenotype correlations in liver. Colors (n=9) on the left represent clusters of co-expressed genes. Numbers in each box report the correlation (top number) and its significance (bottom number, in parenthesis) between each cluster and the phenotypes listed across the bottom. Tested phenotypes were based on the information in Supplementary Material, Supplementary Table 1.

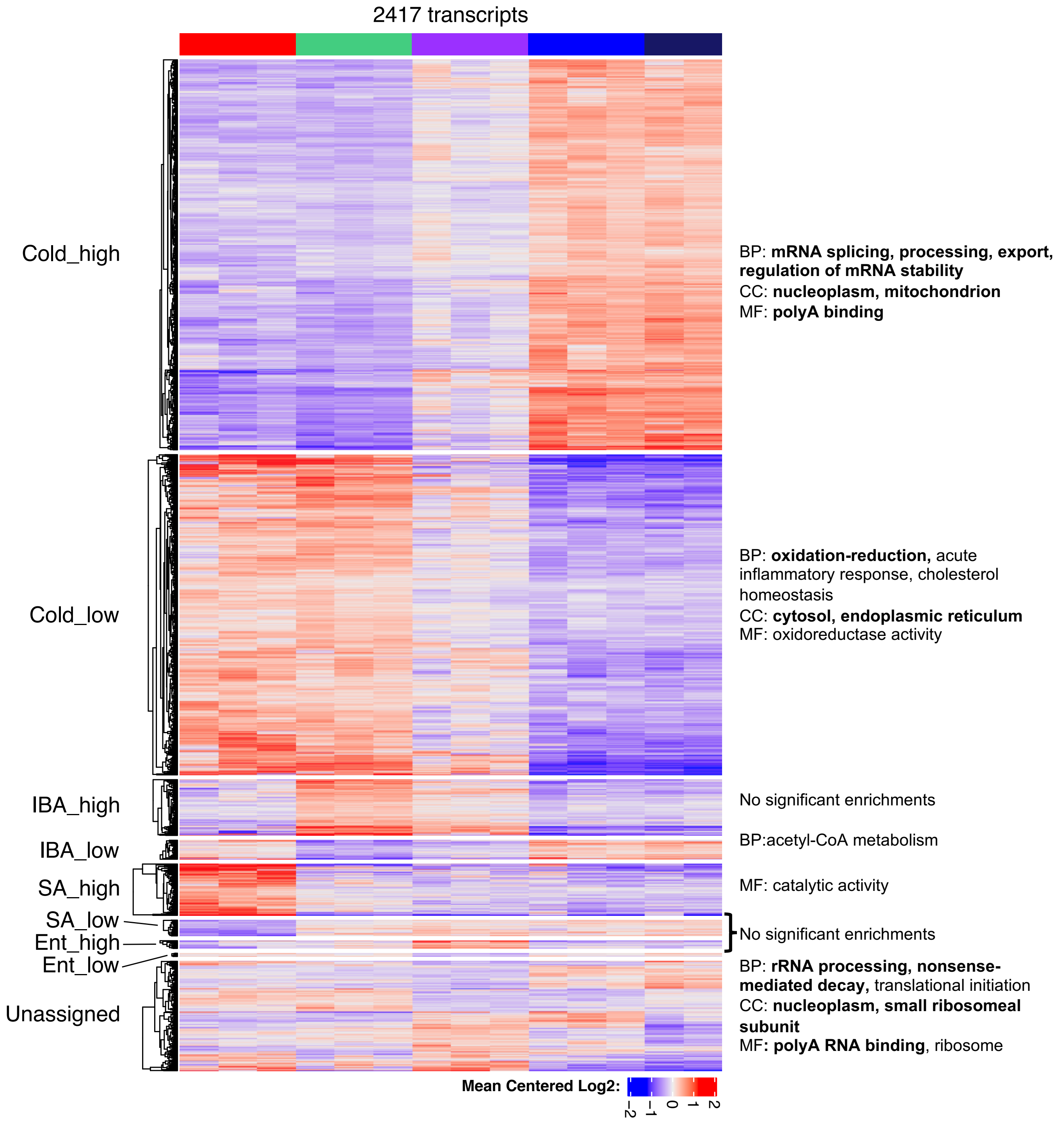

Supplementary Figure 5. Heatmap of GRO-seq DE genes, clustered by expression pattern. Top gene enrichments for each cluster are indicated on the right for GO terms Biological Process (BP), Cell Component (CC) and Molecular Function (MF), in bold if q<0.001, otherwise p<0.001. See also Figure 5, Supplementary Table 4.

A) SCD_containing, seasonally DE (RNA-seq) and concordant between RNA-seq and GRO-seq

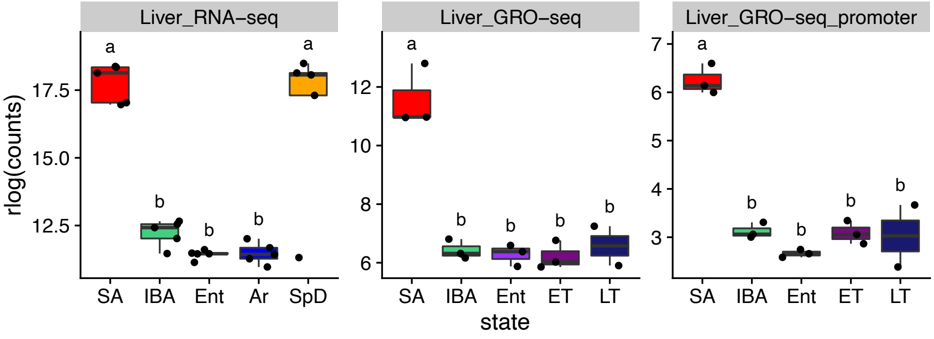

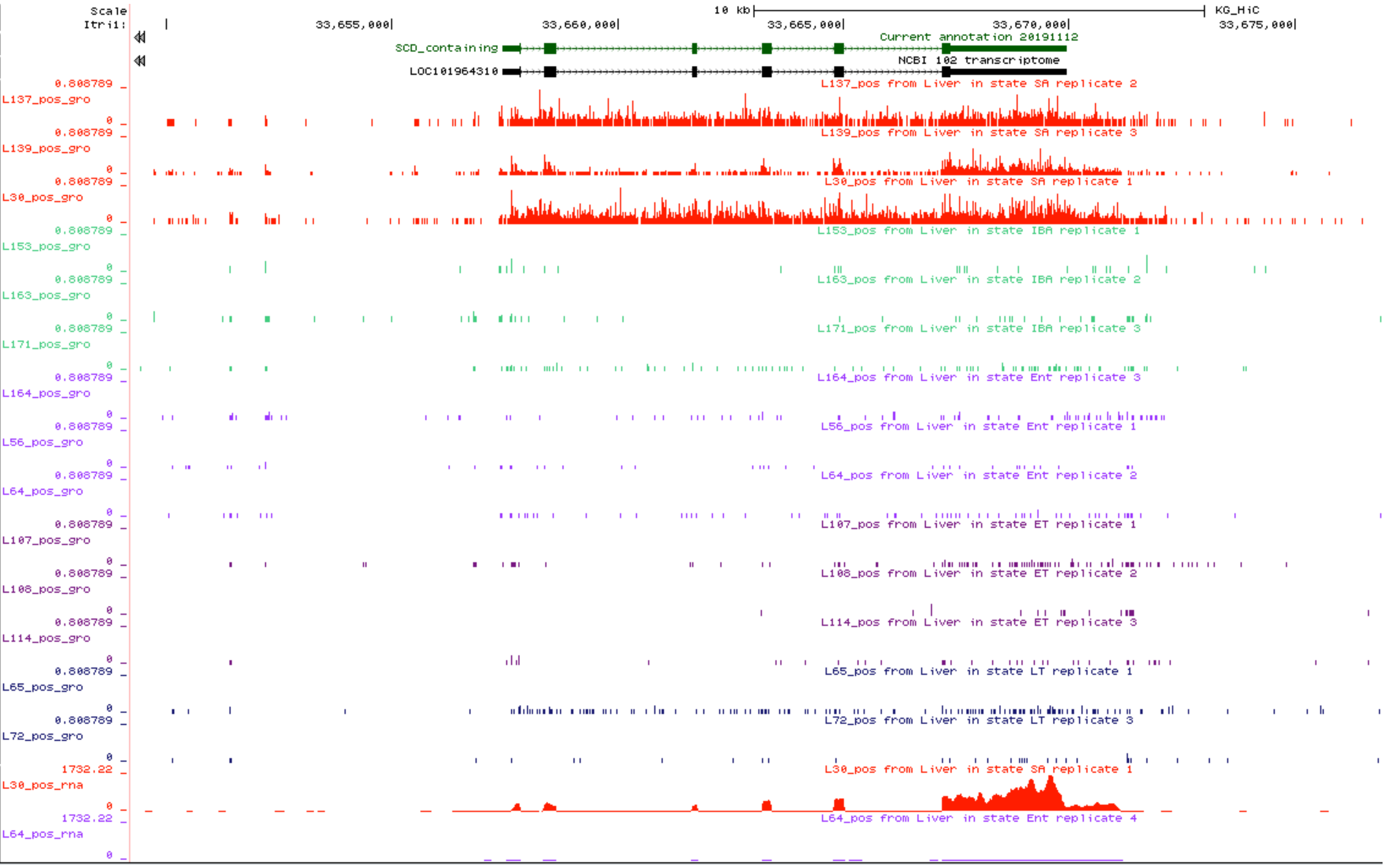

B) Arrdc3, DE in torpor-arousal cycle (RNA-seq), concordant between RNA-seq and GRO-seq

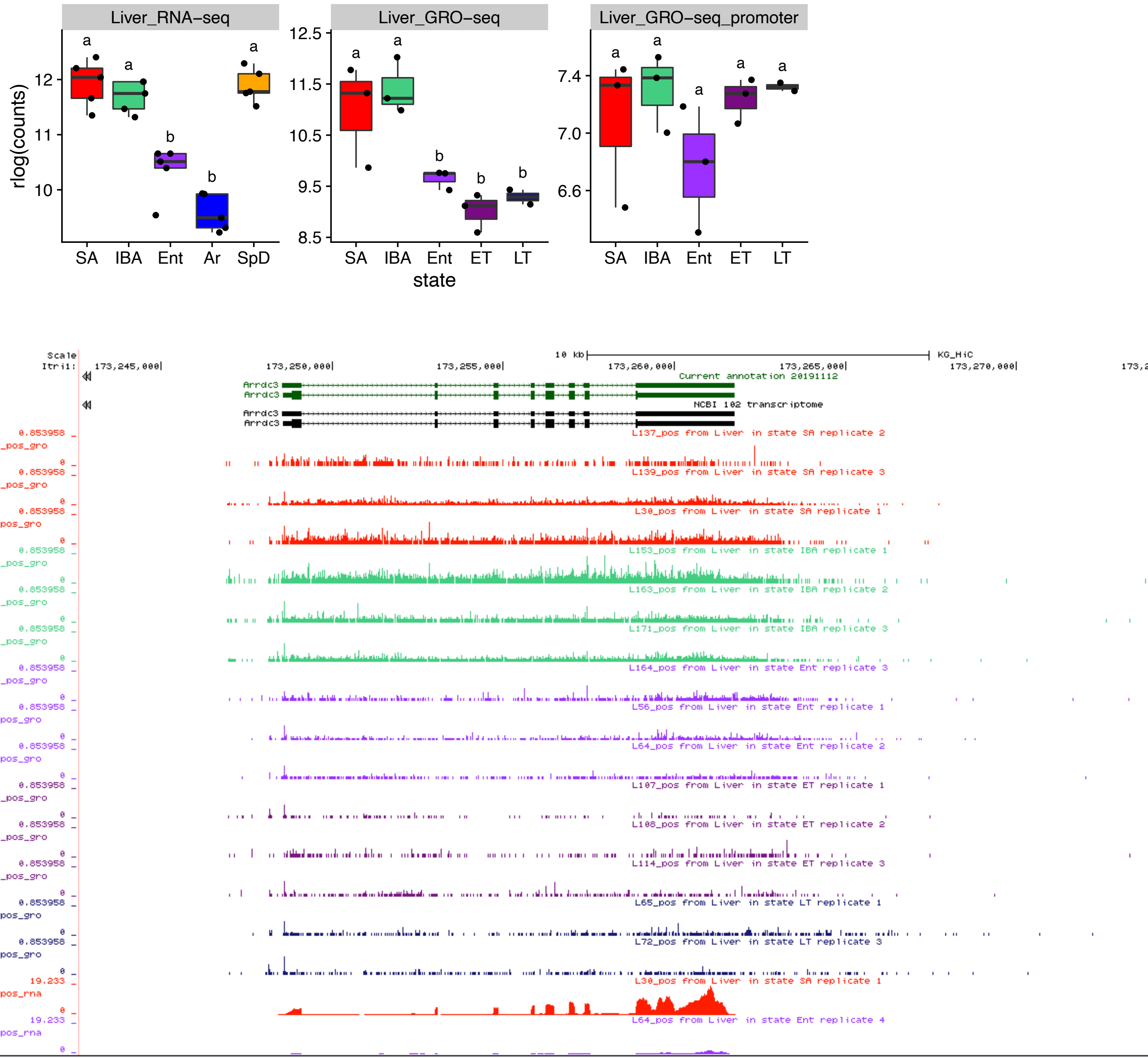

C) SerpinA7, DE in torpor-arousal cycle (RNA-seq), discordant between RNA-seq and GRO-seq

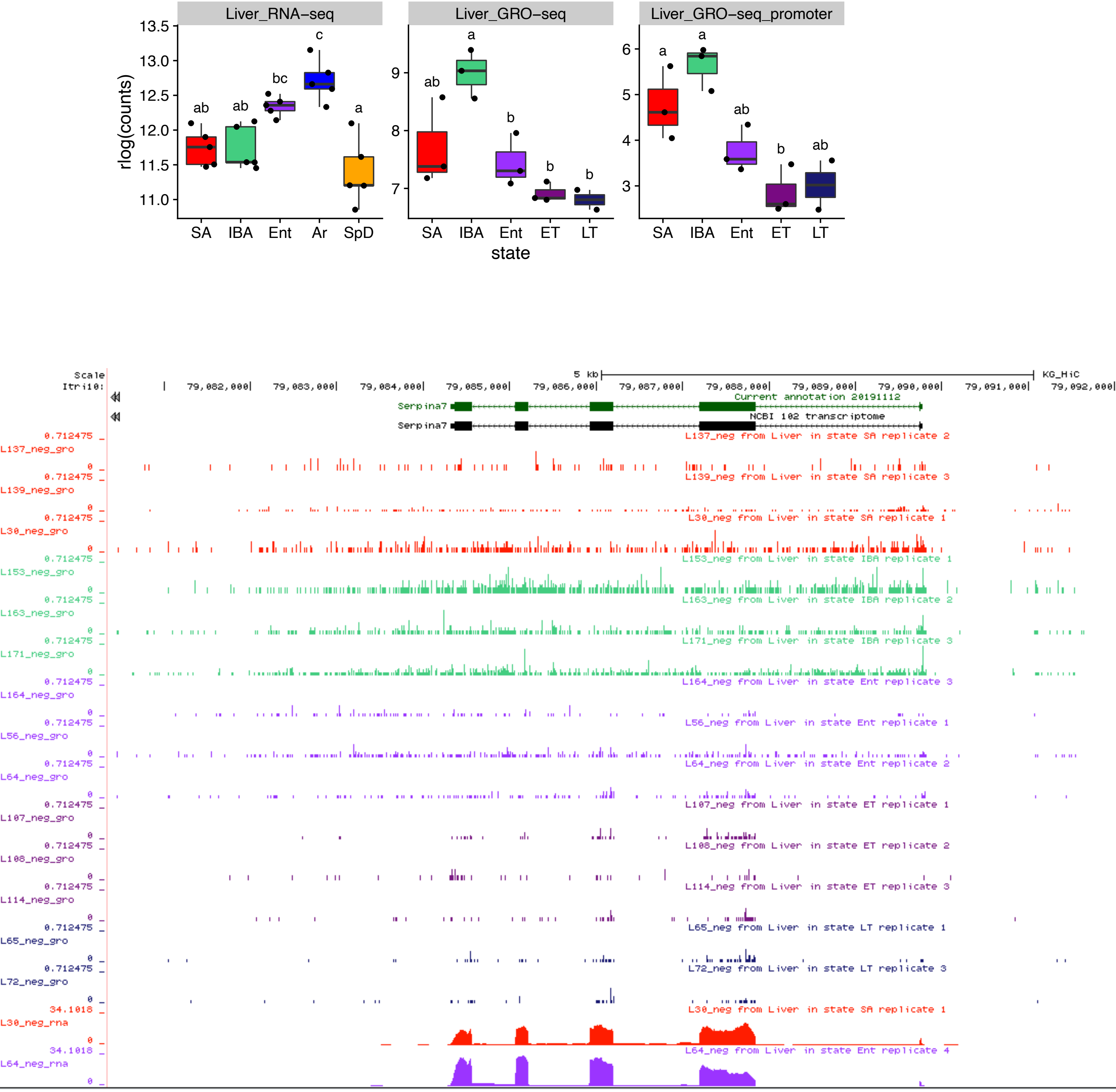

D) A2m, DE seasonally and across torpor-arousal cycle (RNA-seq), discordant between RNA-seq and GRO-seq

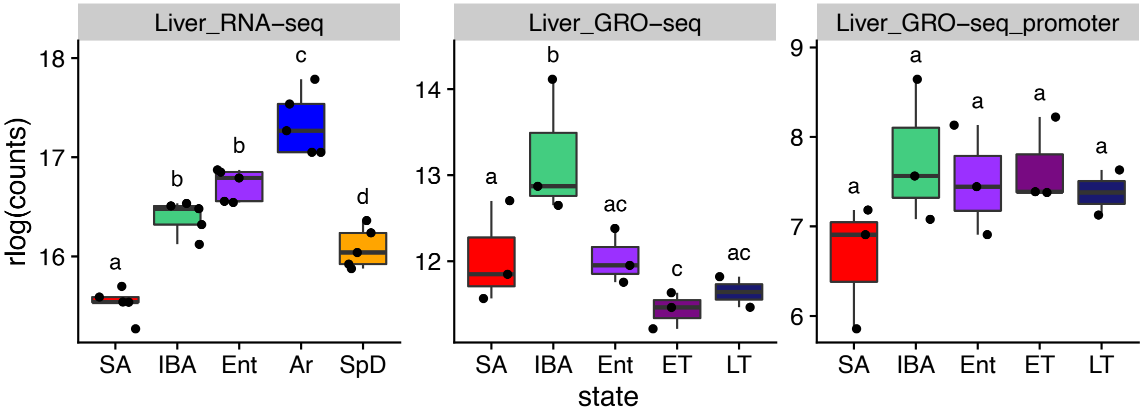

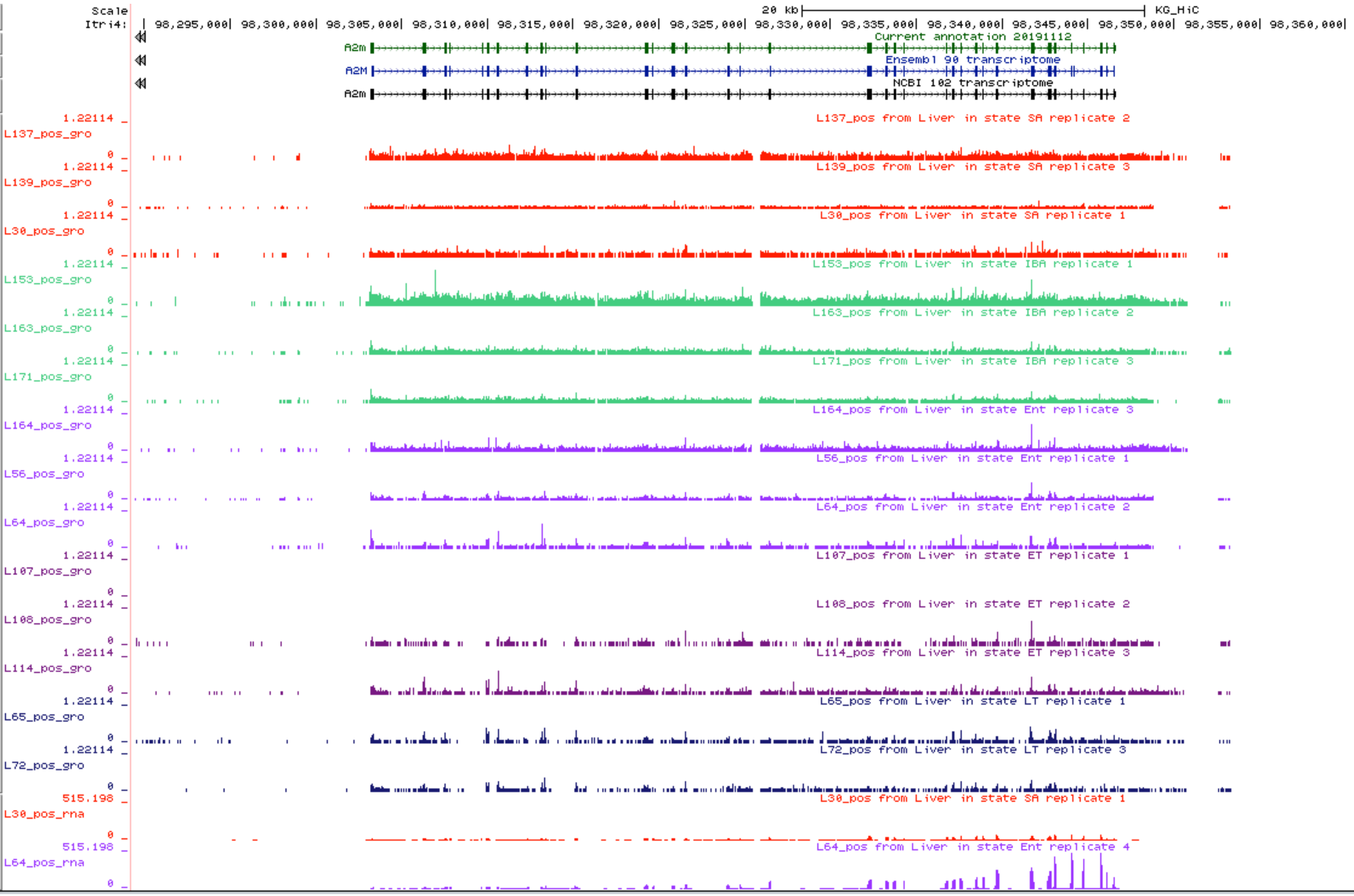

E) Hsp90aa1: DE in torpor-arousal cycle (RNA-seq), discordant between RNA-seq and GRO-seq

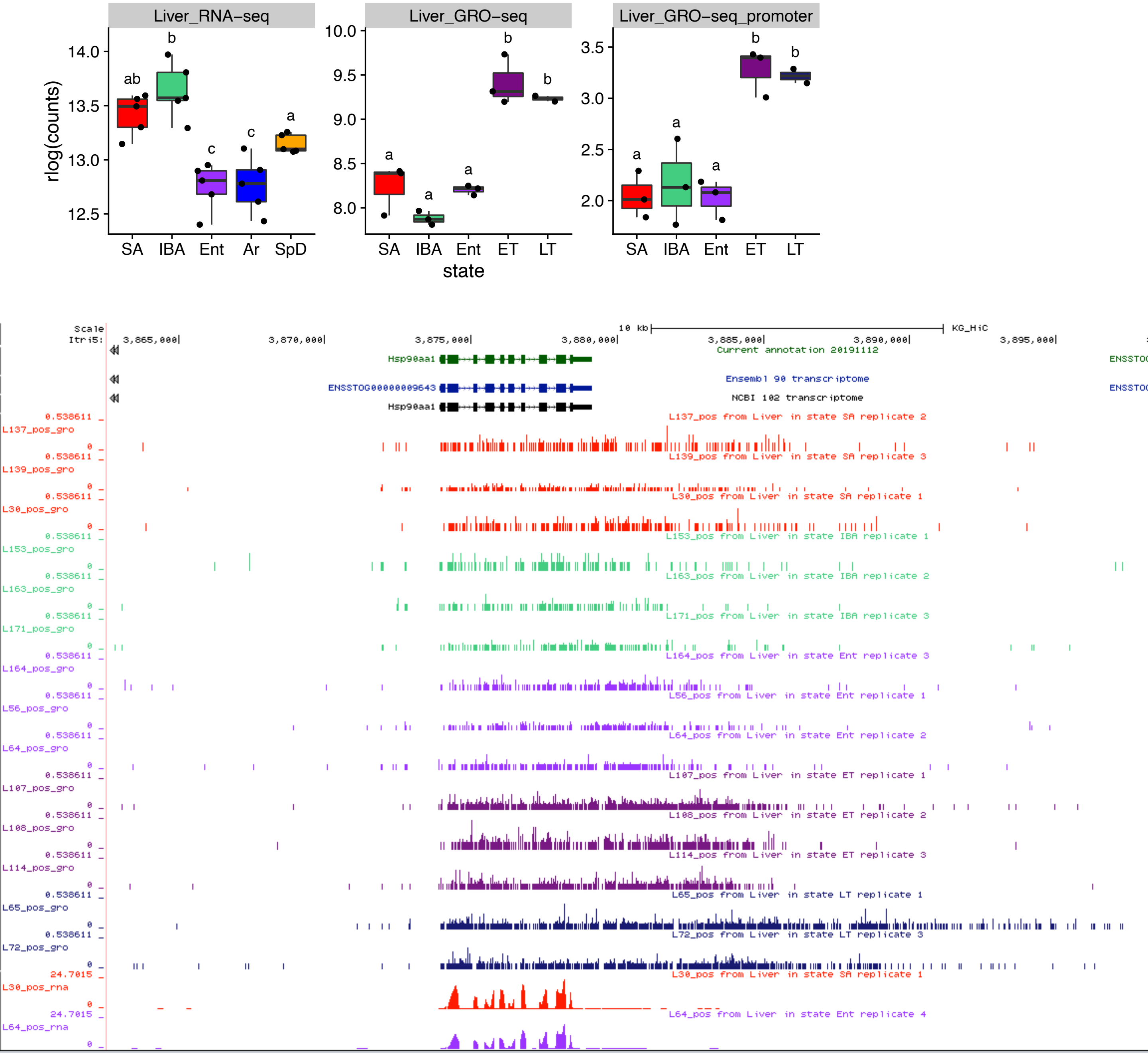

F) Atp6v1b2: DE in torpor-arousal cycle (RNA-seq), discordant between RNA-seq and GRO-seq

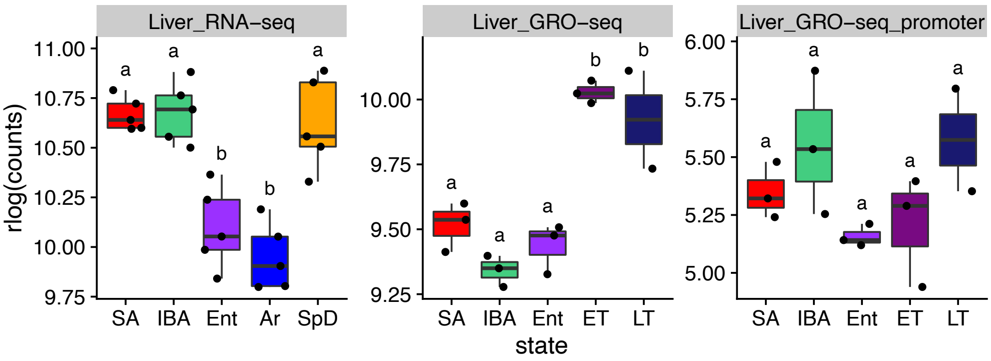

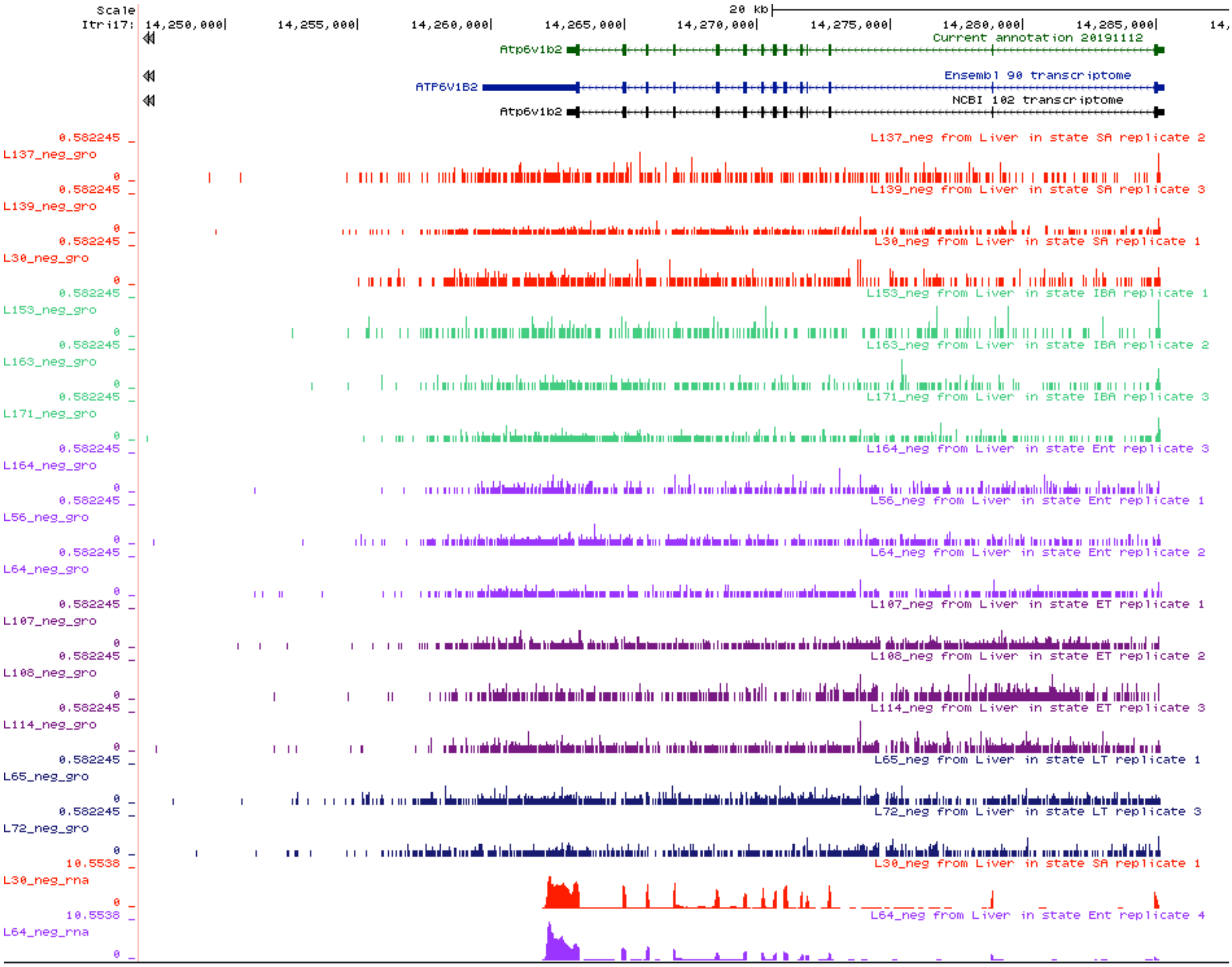

Supplementary Figure 6. Complex gene expression dynamics in the torpor-arousal cycle revealed by comparison of GRO-seq and RNA-seq data. For each gene in panels A-F, boxplots (squirrelBox) show relative abundance changes in the liver steady-state RNA (liver_RNA-seq), and transcription across the gene body (Liver_GRO-seq, fstitch extended annotations excluding first 500nt) and in the promoter region (Liver_GRO-seq_promoter, first 500nt of gene). The accompanying gene browser views show regions with relevant signal surrounding the individual annotated gene in the Itri_HiC_2 genome with all GRO-seq tracks and the two common RNA-seq sample tracks for the transcribed strand. Example concordant and discordant genes between RNA-seq and GRO-seq are A) SCD_containing_16206, B) Arrdc3, C) SerpinA7, D) A2m, E) Hsp90aa1, F) Atp6v1b2. See also Figure 6, Supplementary Table 5.

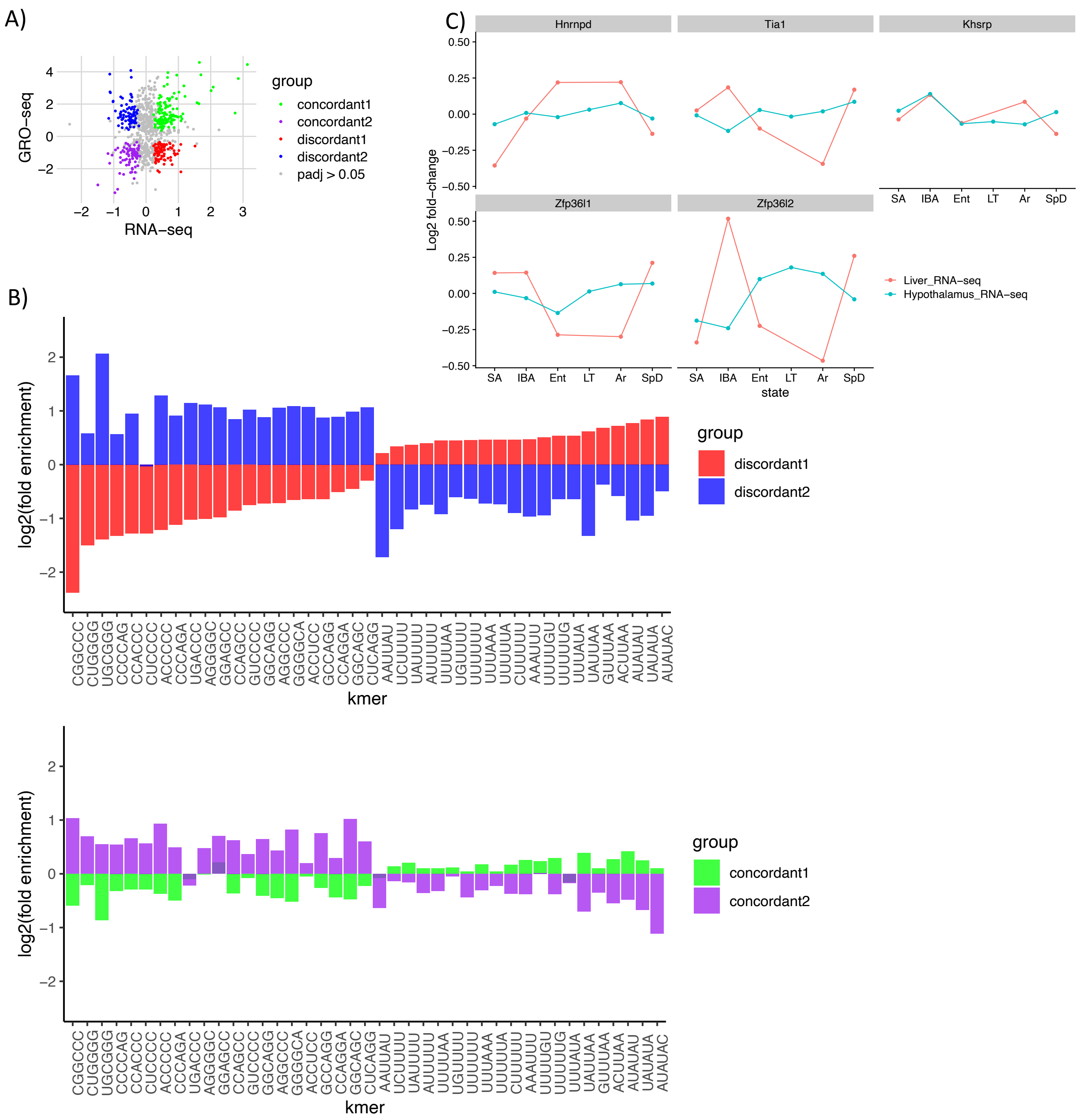

Supplementary Figure 7. Sequence features that alter RNA stability and utilization. A) Scatterplot of correlations between RNA-seq and GRO-seq data in the IBA vs. Ar (RNA-seq) or LT (GRO-seq) pairwise comparisons. Each dot is one gene, colors indicate group designations based on whether abundance was increased or decreased in IBA compared to Ar or LT in the RNA-seq or GRO-seq dataset, respectively. q<0.05 was used, relaxed compared to q<0.001 elsewhere, to capture more genes for motif analysis. B) Distinct motif enrichments in 3' NC region of genes depending on the group memberships depicted in panel A. Enriched motifs in the 3'UTR were analyzed using the transite R package. Motifs with adjusted p value <= 0.001 enrichment and depletion are plotted, arranged by log2 fold enrichment in discordant group 1. C) Lineplots show largely distinct gene expression dynamics for DE transcripts encoding ARE-binding proteins in hibernating liver compared to hypothalamus.

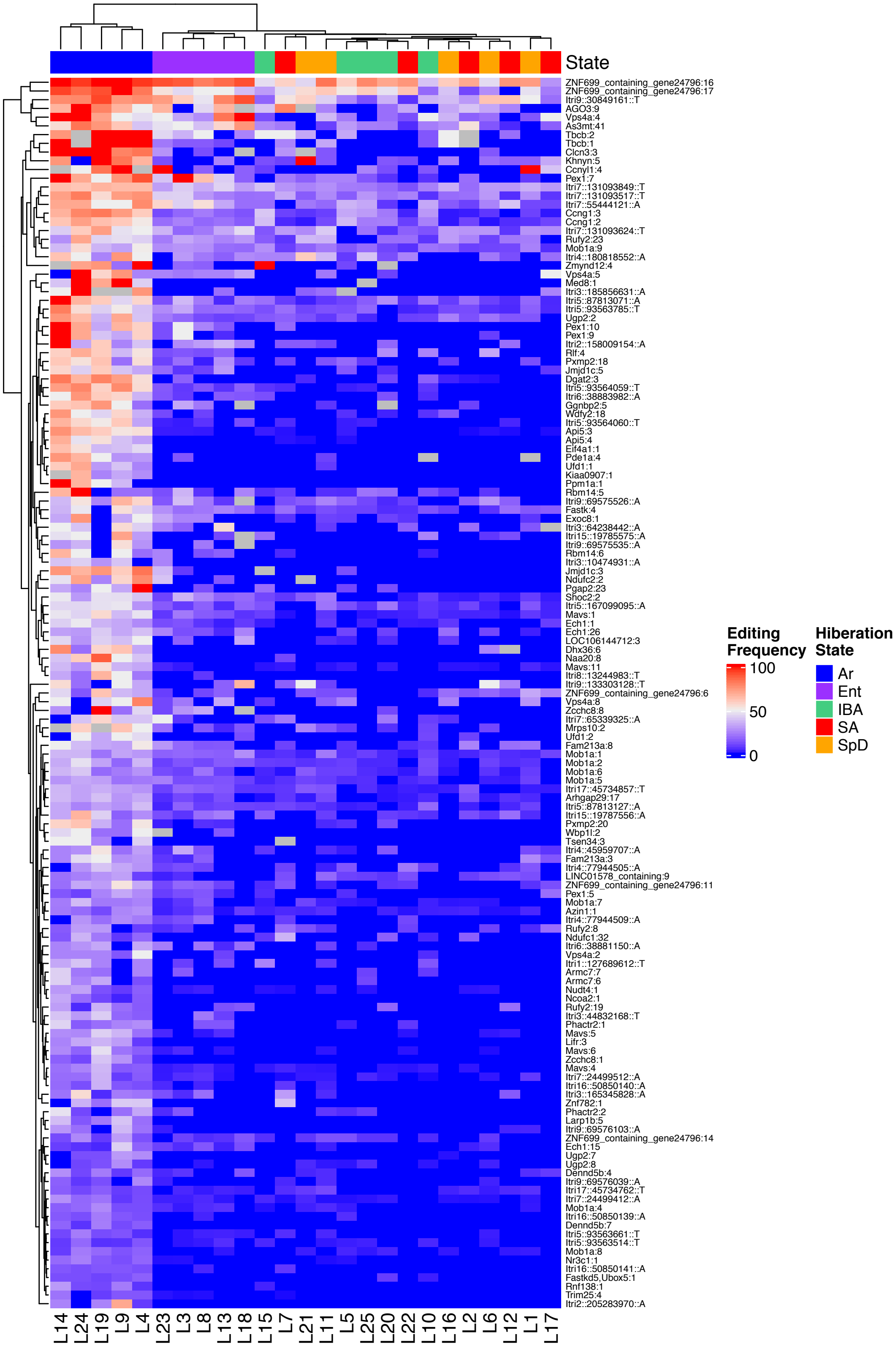

Supplementary Figure 8. Heatmap of editing frequencies of cold-enriched A-to-I editing sites. Liver editing frequencies based on RNA-seq data are plotted for genes that were also edited in brain (Riemondy et al., 2018). Editing sites are denoted as the gene or genes overlapping each editing site and either A or T if the editing site is on the positive or negative strand. Intergenic sites are indicated by contig and reference position. See also Supplementary Table 6.

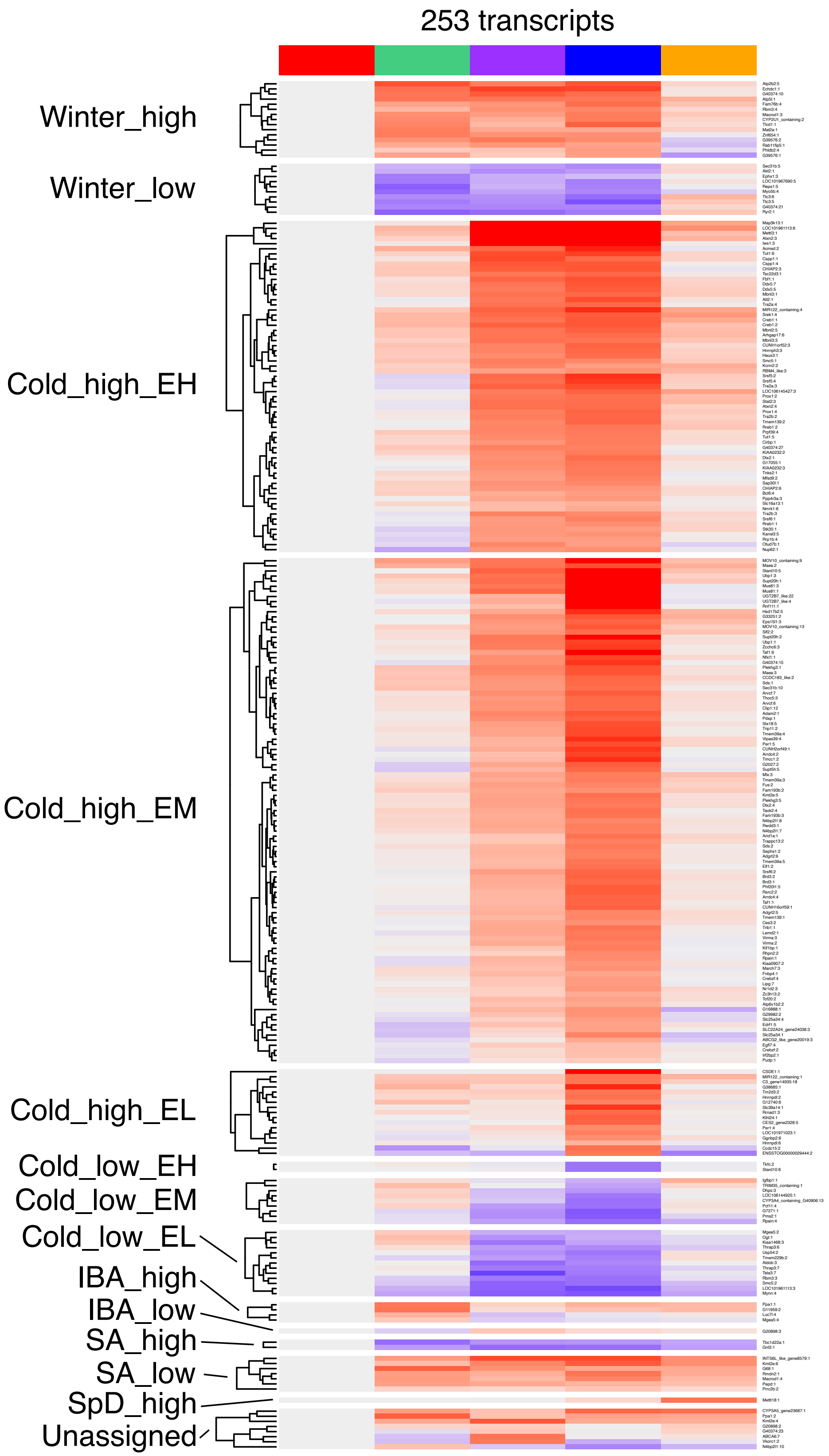

Supplementary Figure 9. Genes with alternative splice sites in liver. Heatmap of alternatively spliced LSVs clustered by pattern, each row represents one gene. For each LSV, the abundance of all junctions except the one most commonly observed in SA is plotted relative to SA. dPSI > 0 is increased alternative splicing relative to SA (alternative junction becomes more common), while dPSI < 0 indicates decreased alternative splicing relative to SA. Genes with multiple significant LSVs have numeric suffixes appended to their gene name following a colon. See also Supplementary Table 7.

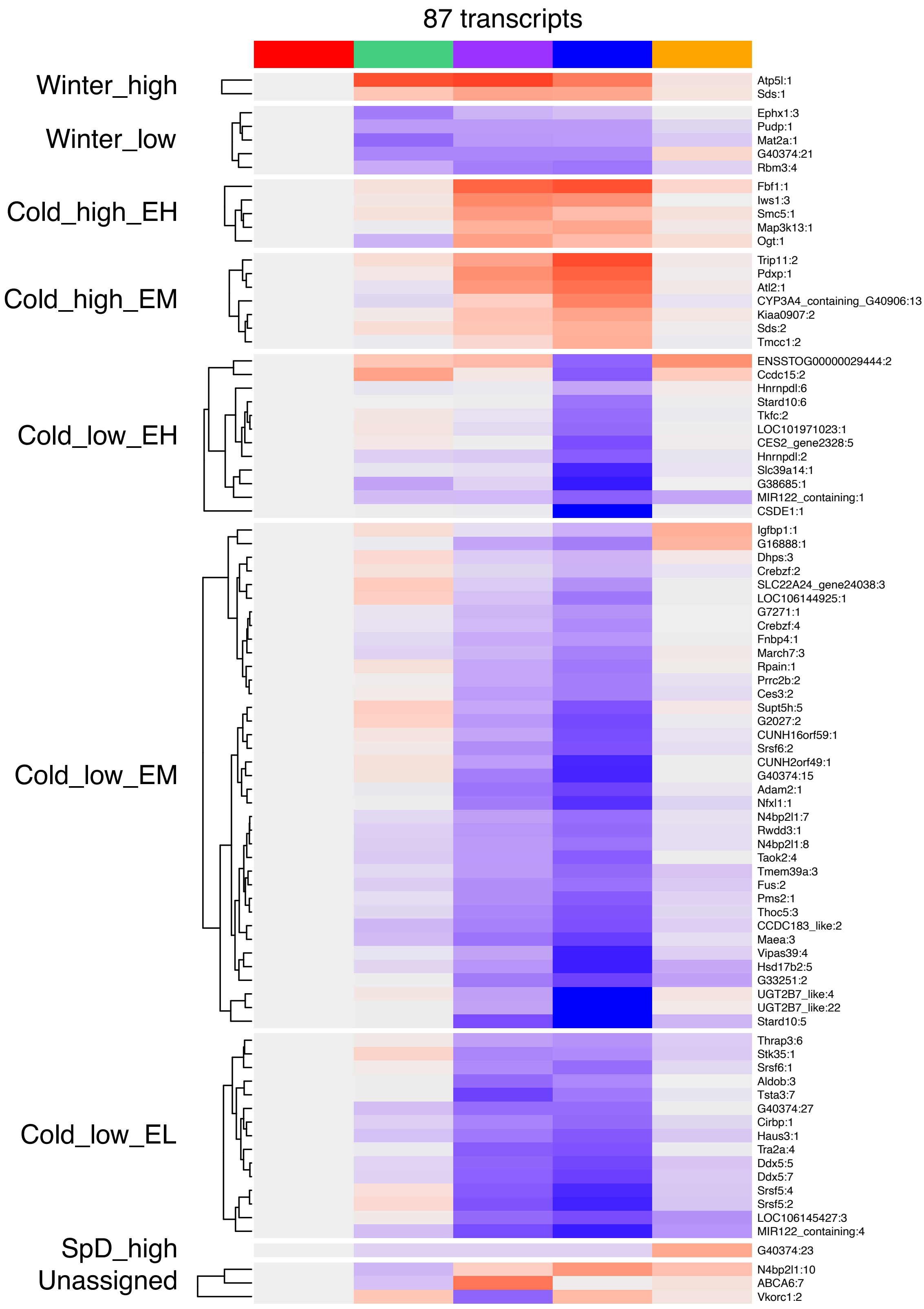

Supplementary Figure 10. Genes with retained introns in liver. Heatmap depicts the abundance of retained introns for each state relative to SA. dPSI > 0 means increased retention of the intron relative to SA, while dPSI < 0 means increased excision of the intron relative to SA. Gene symbols are shown to the right of the heatmap. For genes with multiple LSVs, the number is appended to the gene name, separated by a colon. See also Supplementary Table 7.

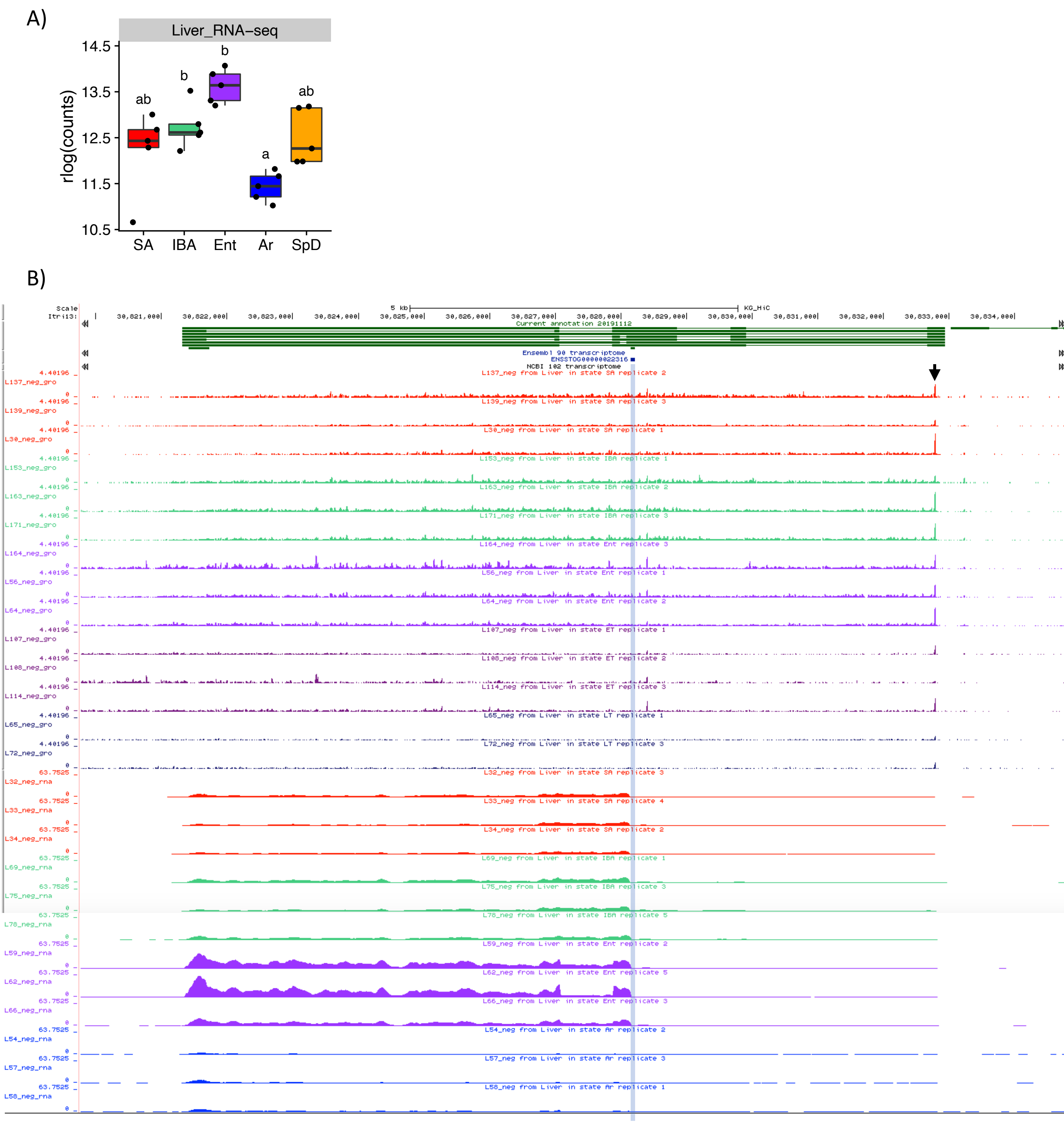

Supplementary Figure 11. GRO-seq and RNA-seq coverage of the MIR122_containing gene. A) Boxplot of RNA-seq quantification of MIR122_containing gene transcripts across physiological states; B) Genome browser screen shots showing distribution of GRO-seq and RNA-seq reads on the MIR122_containing gene. While GRO-seq coverage fell below filtering thresholds for DESeq2 analysis, GRO-seq read coverage is consistent with transcription from the TSS (black arrow where increased read density reflects a promoter-proximal pause) through the 3' end of the gene during SA, IBA and Ent, with reduced transcription during torpor (ET and LT). RNA-seq coverage shows that reads responsible for the quantitative differences of this transcript among groups largely lie 3' of the cleaved miRNA for this minus strand gene (the position of MIR122 is marked by the transparent vertical blue line). The three remaining RNA-seq samples after removing the highest and lowest sample from each group are shown.

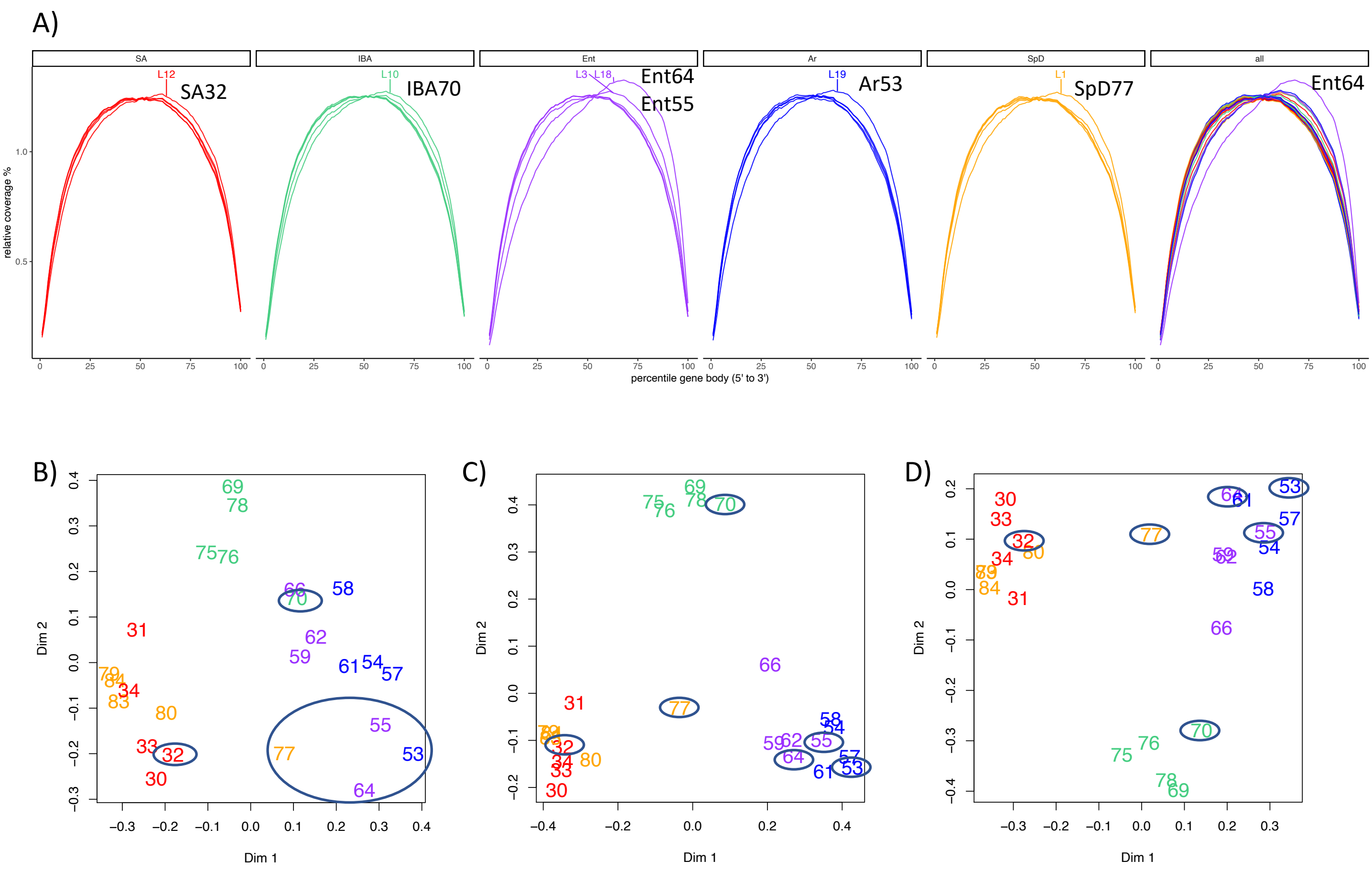

Supplementary Figure 12. Features of RNA-seq data. A) Overall gene body coverage against all genes longer than 100nt in the HiC_Itri_2 genome annotation. Relative coverage percentages were plotted for normalized gene body percentile, with 0 and 100 representing the 5' and 3' ends, respectively. For each state (Figure 1) except Ent, one sample (labeled with animal number in black) exhibited a slight 3' bias compared to coverage across the normalized gene body seen in the remaining samples; a second Ent sample was the most extreme outlier (Ent64, rightmost "all" plot). RIN numbers did not differ among groups (ANOVA pval = 0.82). Animal 64 was one of the two samples used for both RNA-seq and GRO-seq analyses and is thus shown in Supplementary Figure 6 despite not being exemplary or even representative of the RNA-seq data quality. B-D) Two-dimensional scaling plots of unsupervised clustering of all samples by random forest using gene-based transcript abundances for: B) all 10,370 pass- filter genes (data from Figure 2A replotted with animal number shows dominance of physiological state (indicated by color) over 3' bias (circled); C) 3,120 genes identified as DE (q<0.001) using DESeq2; D). The topmost 3,120 genes after ranking the 10,370 pass-filter genes by variance across all 25 samples.

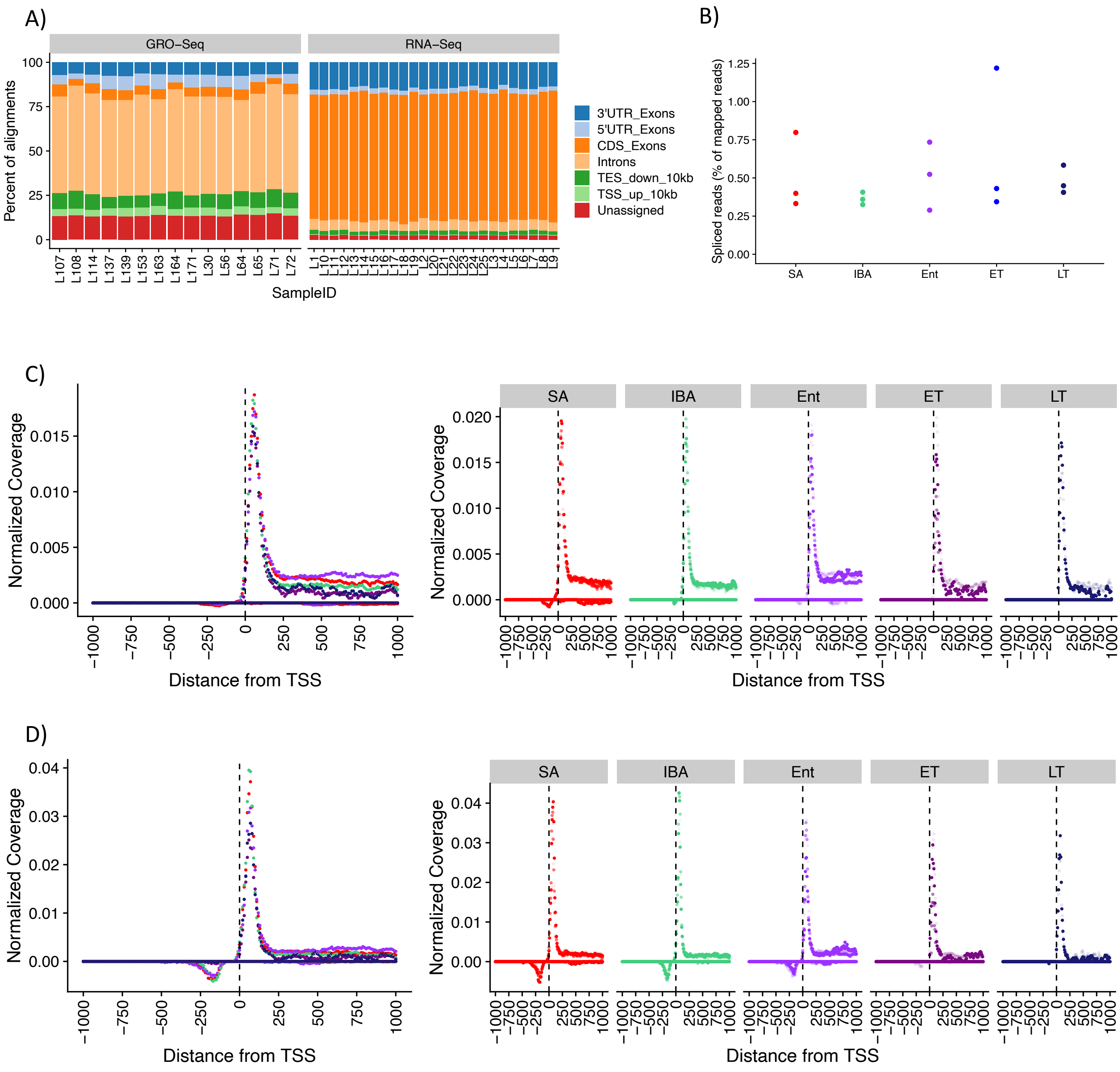

Supplementary Figure 13. Features of GRO-seq data. A) Bargraphs plot the relative distribution of sequence reads over gene features in GRO-seq vs. RNA-seq data; TES_down_10kb is transcript end site plus 10kb, whereas TSS_up_10kb is the transcript start site plus 10kb upstream. Reads falling outside of these gene boundaries or overlapping more than one gene's annotation are excluded (Unassigned). B) A small fraction of GRO-seq reads are spliced in all libraries; each dot represents one sample. C, D) Metagenes of GRO-seq read coverage near the transcription start site were constructed by defining genomic intervals as described in methods, from C) 533 genes, or D) after selecting the 124 genes with bidirectional fstitch annotations surrounding the annotated TSS.
