# Supplementary material for: Liver Transcriptome Dynamics During Hibernation Are Shaped by a Shifting Balance Between Transcription and RNA Stability
Source: Front Physiol. 2021 May 21;12:662132. doi: 10.3389/fphys.2021.662132 (PMC8176218; doi:10.3389/fphys.2021.662132)
Supplement: Supplementary file 2 [file Data_Sheet_2.PDF]

Supplementary Materials and Table Legends

List of Supplementary Materials

Supplementary Datasheet 1. Supplementary Figures 1-13.
Supplementary Datasheet 2. List of all Supplementary Materials, Supplementary Table Legends (this document).
Supplementary Datasheet 3. Full DESeq2 output for RNA-seq dataset, supports Supplementary Tables 2 and 5.
Supplementary Datasheet 4. Full DESeq2 output for GRO-seq dataset using Fstitch annotation, eliminating reads mapping to first 500nt of annotated transcripts and overlapping genes, supports Supplementary Tables 4 and 5.
Supplementary Datasheet5. GRO-seq read count matrix for reannotation, supports Supplementary Datasheet 4, Supplementary Tables 4 and 5.

Supplementary Table 1. Metadata. Excel file with four tabs.
Supplementary Table 2. Differentially-expressed genes in hibernating liver. Excel file with 7 tabs.
Supplementary Table 3. Gene enrichments in liver DE genes by cluster. Excel file with 2 tabs.
Supplementary Table 4. Differentially-transcribed genes in hibernating liver. Excel file with 6 tabs.
Supplementary Table 5. Genes DE in both RNA-seq and GRO-seq datasets. Excel file with 7 tabs.
Supplementary Table 6. Summary of ADAR-edited sites in liver RNA across pairs of physiological states. Excel file with one tab.
Supplementary Table 7. Gene enrichments found in alternative spliced liver genes, excel file with 2 tabs.

Supplementary Table Legends

Supplementary Table 1. Metadata, excel file with 4 tabs with sequencing and mapping characteristics for RNA-seq and GRO-seq libraries, and animal data for the two library sets. Two samples, SA30 and Ent64 were obtained from the same animal and used for both RNA-seq and GRO-seq (numbers indicated in red). The GRO-seq sample, LT71 (row indicated by gray type), was of relatively poor quality. It was removed from all further analyses after it was also identified as an outlier by PCA.

Supplementary Table 2. Differentially-expressed (DE) genes across hibernation states identified by DESeq2 analysis of RNA-seq data. Seven tabs contain full information for DESeq2 analysis of liver RNA-seq data for all DE genes (Liver_DE tab), plus six tabs with pairwise DE genes including just the gene name, fold change (two methods), wald_pvalue and wald_padj for the indicated pair (supports Figure 2).

Supplementary Table 3. Gene enrichments returned by DAVID (Huang et al., 2009) in gene co-expression clusters based on RNA-seq data (supports Supplementary Figure 2) or GRO-seq data

as indicated. All enrichments p<0.01 are given, those with q<0.001 are indicated by boldface (supports Supplementary Figures 2 and 5).

Supplementary Table 4. Differentially-transcribed genes across hibernation states identified by DESeq2 analysis of GRO-seq data in non-overlapping gene bodies. Six tabs contain full information for DESeq2 analysis for all differentially-transcribed genes (GROseqAll_sig001), plus five tabs with fold change and significance data for genes differentially-transcribed in all possible sequential pairwise transitions among physiological states (supports Figure 5).

Supplementary Table 5. Genes both DE in RNA-seq and GRO-seq by DESeq2. All genes are listed on GandR001 tab, then 6 additional tabs with the indicated pairwise comparisons, with IBA compared to either Ar (RNA-seq) or LT (GRO-seq) broken into concordant and discordant groups (supports Figure 6).

Supplementary Table 6. RNA-editing analysis of liver RNA-seq data for each site (supports Figure 7, Supplementary Figure 8).

Supplementary Table 7. Gene enrichment categories in the alternatively spliced liver genes. Two tabs show all GO term enrichments for all genes and by cluster as indicated.
